# Neuroinvasive *Listeria monocytogenes* Infection Triggers IFN-Activation of Microglia and Upregulates Microglial miR-155

**DOI:** 10.3389/fimmu.2018.02751

**Published:** 2018-11-27

**Authors:** Miao Zhang, Allison. F. Gillaspy, Jenny R. Gipson, Benjamin R. Cassidy, Jessica L. Nave, Misty F. Brewer, Julie A. Stoner, Jie Chen, Douglas A. Drevets

**Affiliations:** ^1^Department of Medicine, University of Oklahoma Health Sciences Center, Oklahoma City, OK, United States; ^2^Department of Microbiology and Immunology, University of Oklahoma Health Sciences Center, Oklahoma City, OK, United States; ^3^Laboratory for Molecular Biology and Cytometry Research, University of Oklahoma Health Sciences Center, Oklahoma City, OK, United States; ^4^Department of Biostatistics and Epidemiology, University of Oklahoma Health Sciences Center, Oklahoma City, OK, United States; ^5^Histology and Immunohistochemistry Core, Peggy and Charles Stephenson Cancer Center, University of Oklahoma Health Sciences Center, Oklahoma City, OK, United States; ^6^Department of Veterans Affairs Medical Center, Oklahoma City, OK, United States

**Keywords:** microRNA, meningitis, brain inflammation, microglia, interferon, *Listeria*

## Abstract

MicroRNA (miR) miR-155 modulates microglial activation and polarization, but its role in activation of microglia during bacterial brain infection is unclear. We studied miR-155 expression in brains of C57BL/6 (B6.WT) mice infected i.p. with the neuro-invasive bacterial pathogen *Listeria monocytogenes* (*L. monocytogenes*). Infected mice were treated with ampicillin starting 2 days (d) post-infection (p.i.) and analyzed 3d, 7d, and 14d p.i. Virulent *L. monocytogenes* strains EGD and 10403s upregulated miR-155 in whole brain 7 d p.i. whereas infection with avirulent, non-neurotropic Δ*hly* or Δ*actA L. monocytogenes* mutants did not. Similarly, infection with virulent but not mutated bacteria upregulated IFN-γ mRNA in the brain at 7 d p.i. Upregulation of miR-155 in microglia was confirmed by qPCR of flow cytometry-sorted CD45^int^CD11b^pos^ brain cells. Subsequently, brain leukocyte influxes and gene expression in sorted microglia were compared in *L. monocytogenes*-infected B6.WT and B6.Cg-Mir155tm1.1Rsky/J (B6.miR-155^−/−^) mice. Brain influxes of Ly-6C^high^ monocytes and upregulation of IFN-related genes in microglia were similar to B6.WT mice at 3 d p.i. In contrast, by d 7 p.i. expressions of microglial IFN-related genes, including markers of M1 polarization, were significantly lower in B6.miR-155^−/−^ mice and by 14 d p.i., influxes of activated T-lymphocytes were markedly reduced. Notably, CD45^high^CD11b^pos^ brain cells from B6.miR-155^−/−^ mice isolated at 7 d p.i. expressed 2-fold fewer IFN-γ transcripts than did cells from B6.WT mice suggesting reduced IFN-γ stimulation contributed to dampened gene expression in B6.miR-155^−/−^ microglia. Lastly, *in vitro* stimulation of 7 d p.i. brain cells with heat-killed *L. monocytogenes* induced greater production of TNF in B6.miR-155^−/−^ microglia than in B6.WT microglia. Thus, miR-155 affects brain inflammation by multiple mechanisms during neuroinvasive *L. monocytogenes* infection. Peripheral miR-155 promotes brain inflammation through its required role in optimal development of IFN-γ-secreting lymphocytes that enter the brain and activate microglia. Microglial miR-155 promotes M1 polarization, and also inhibits inflammatory responses to stimulation by heat-killed *L. monocytogenes*, perhaps by targeting *Tab2*.

## Introduction

Severe bacterial infections such as meningitis and sepsis trigger CNS inflammatory pathways that are key drivers of neurologic damage and dysfunction (1–3). Corticosteroids are the principal means used in clinical practice to ameliorate brain inflammation during bacterial meningitis (1). This treatment clearly benefits some patients, but recent analyses show it does not reduce mortality and severe neurological sequelae in all groups of patients (4–6). Moreover, corticosteroid treatment does not improve long-term neurological outcomes in survivors of bacterial meningitis (7). Thus, there is a compelling need to develop new forms of adjunctive therapy that reduce CNS inflammation caused by bacterial infections (8).

Activated microglia and the pro-inflammatory mediators they produce can affect cognitive function or cause mood disorders, and are key contributors to poor outcomes in bacterial meningitis and sepsis (8–10). Recent studies show that microRNA (miR) such as miR-124, miR-146a, and miR-155 highly influence microglial activation and polarization (11, 12). Importantly, manipulating microglial miR expression in animal models of inflammatory diseases of the CNS can have salutary effects suggesting a potential for exerting similar benefits when CNS inflammation is triggered by bacterial infection (13).

miR are short, non-coding RNA that influence many different cellular processes via post-transcriptional regulation of mRNA (14, 15). miR alter inflammation in part by modulating TLR signaling, transcription factor expression, and cytokine production (16). miR-155 and miR-146a are upregulated via NFκB in response to TLR-signaling and to cytokines such as IFN-β, IFN-γ, and TNF (16, 17). miR-155 is upregulated *in vivo* by experimental middle cerebral artery occlusion, multiple sclerosis, the SOD1 model of amyotrophic lateral sclerosis, and Japanese encephalitis virus infection (13, 18–20). In microglia, miR-155 is upregulated during M1-polarization and promotes inflammation, whereas miR-146a inhibits inflammation via negative regulation of NFκB signaling (21, 22). In contrast, miR-124a is expressed in resting microglia and is down-regulated by classical (LPS) and alternative (IL-4) polarizing stimuli (23). Expression of miR-124 skews microglial polarization through down-regulation of PU.1-mediated cell differentiation via direct inhibition of C/EBPα transcription factor expression (23).

Because each miR can interact with many different mRNAs, a given miR can cause divergent effects, e.g., pro-inflammatory or anti-inflammatory, in different cells or under different conditions (24). For example, miR-155 induces inflammation by stabilizing TNF or by down-regulating mRNA for anti-inflammatory molecules such as phosphatidylinositol-3,4,5-trisphosphate 5-phosphatase 1 (SHIP-1) and suppressor of cytokine signaling (SOCS1) (25–27). In contrast, miR-155 can also inhibit inflammation by targeting components of the NFκB complex (28, 29), down-regulating MyD88 (30, 31), targeting mRNA for the transforming growth factor-β-activated kinase 1-binding protein 2 (TAB2) adaptor molecule in the TLR/IL-1 signaling pathway, or by targeting *SMAD2* and decreasing IL-1β production (32, 33). miR-155 is also required for optimal development of IFN-γ secreting antigen specific CD8 positive T-cells after viral and bacterial infection (34, 35).

The expression and function of miR-155 in the CNS during bacterial infection is not well-studied. We hypothesized that microglial miR-155 had a demonstrable role in brain inflammation during *Listeria monocytogenes* (*L. monocytogenes*) infection. *L. monocytogenes* is a foodborne, facultative intracellular bacterium that causes severe diseases, e.g., sepsis and CNS infections (36). Recent epidemiologic studies show invasive *L. monocytogenes* has an average case-fatality rate of 21% (37), with CNS infections causing fatal disease in 30–36% of cases (38, 39). Moreover, long-term neurological sequelae have been identified in 26–44% of neurolisteriosis survivors (38, 39). The use of corticosteroids as adjunctive treatment in neurolisteriosis is controversial with recent studies showing they confer no benefit, or suggesting a deleterious effect (38, 39).

Experiments reported here studied miR-155 expression in the brains of *L. monocytogenes*-infected mice that were treated with antibiotics to mimic patients treated for invasive listeriosis. In this model, mice were infected with a lethal inoculum of *L. monocytogenes* then treated with ampicillin, the same antibiotic used in humans (36). These studies confirm miR*-*155 is upregulated in microglia in response to neuroinvasive infection. Interestingly, miR*-*155 does not increase during microglial activation triggered by the innate immune response, but occurs later during the adaptive response to infection. Analysis of mRNA expression in microglia shows Type I IFN exerts major effects during the innate response then is supplanted by Type II IFN during the adaptive response. Comparison of responses in C57BL/6 (B6.WT) and B6.Cg-*Mir155*^*tm*1.1*Rsky*^/J (B6.miR-155^−/−^) mice reveals that miR-155 modulates CNS infection during neuroinvasive *L. monocytogenes* infection by multiple mechanisms.

## Materials and methods

### Antibodies

Fluorochrome-conjugated mAb directed against specific antigens and isotype-matched control antibodies were purchased from BD Pharmingen (San Diego, CA): Ly-6G (1A8), Biolegend (San Diego, CA): CD3 (17A2), CD11b (M1.70), CD80 (16-10A1), MHCcII (M5/114.15.2), BioRad (Hercules, CA) CD11b 5c6, and eBioscience (San Diego, CA): CD8a (53-6.7), CD45 (30-F11), Ly-6C (HK1.4), IFN-γ (XMG 1.2), TNF (MP6-XT22).

### Bacteria

*L. monocytogenes* strain EGD was originally obtained from P.A. Campbell (40). Strain 10403s was obtained from the American Type Culture Collection (Manassas, VA). Gene deletion mutants constructed from the 10403s parent strain deficient in listeriolysin O (Δ*hly*) DP-L2161 and *act*A (Δ*actA*) DP-L1942 were gifts from D. Portnoy (Univ. of California, Berkeley, CA) (41, 42). Bacteria were stored in brain-heart infusion (BHI) broth (Difco, Detroit, MI) at 10^9^ CFU/ml at −80°C. For experiments, the stock culture was diluted 1:10,000 into BHI and cultured overnight at 37°C with shaking.

### Animal infection and antibiotic treatment

**Figure 1:**
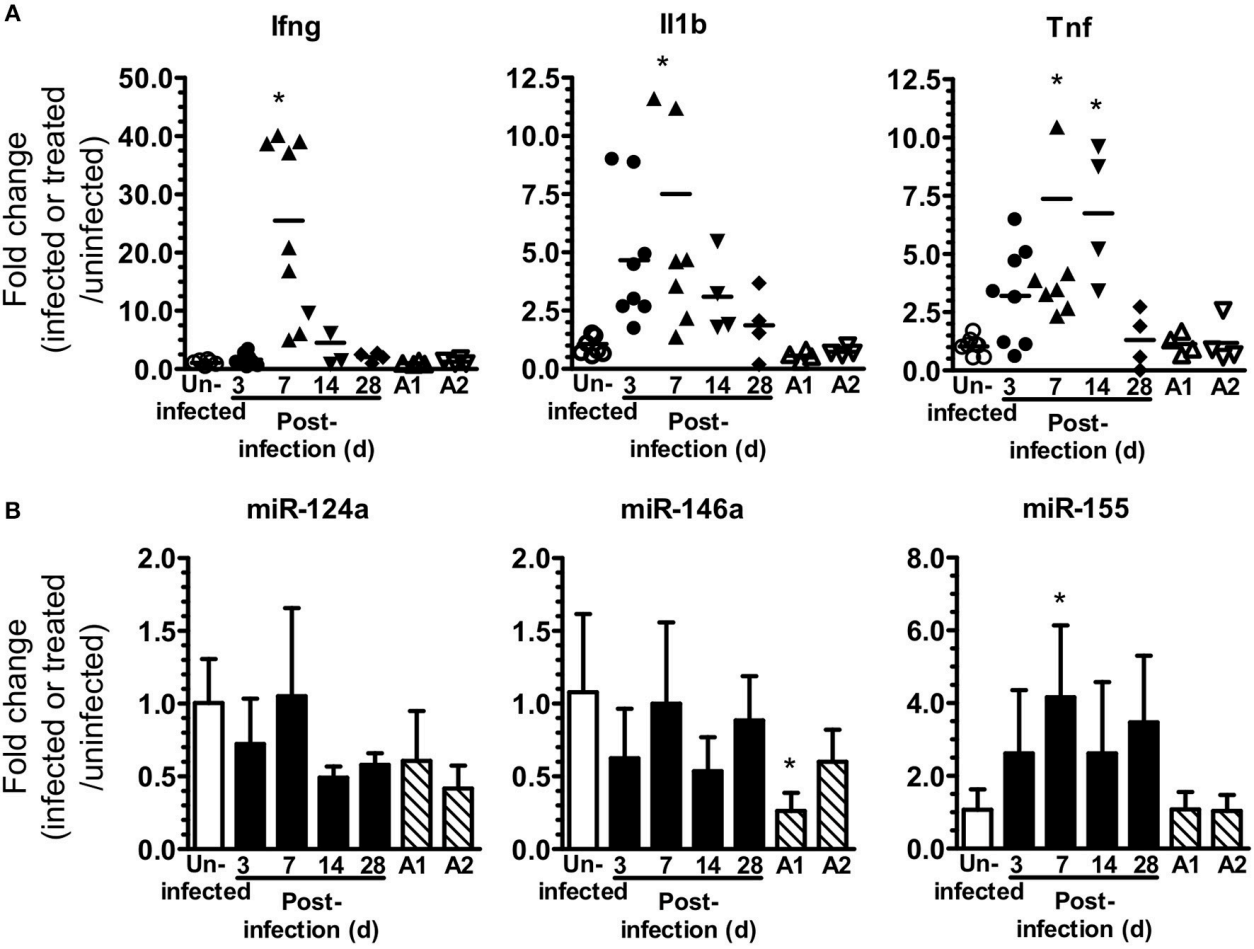
*L. monocytogenes* infection upregulates brain miR-155. **(A,B)** B6.WT mice were infected i.p. with 4.0–4.8 × 10^5^ CFU *L. monocytogenes* EGD and treated with antibiotics. Uninfected mice were neither infected nor treated with antibiotics, whereas other uninfected mice received 3 injections of ampicillin (A1) only, or 3 injections of ampicillin plus oral amoxicillin (A2). Organs were removed after perfusion and expressions of mRNA **(A)** and miR **(B)** were measured by qPCR and normalized to *Gapdh* for mRNA and snoRNA135 for miR. Results from two experiments were combined and are presented as the fold change (infected or treated/uninfected) mRNA expression from individual mice with the bar representing the group mean **(A)**, or mean + SD fold change miR expression from 4 to 8 mice/time point **(B)**. ^*^p < 0.05 compared with same genotype uninfected control by 1-way ANOVA with Dunnett's post-test.

**Figure 2:**
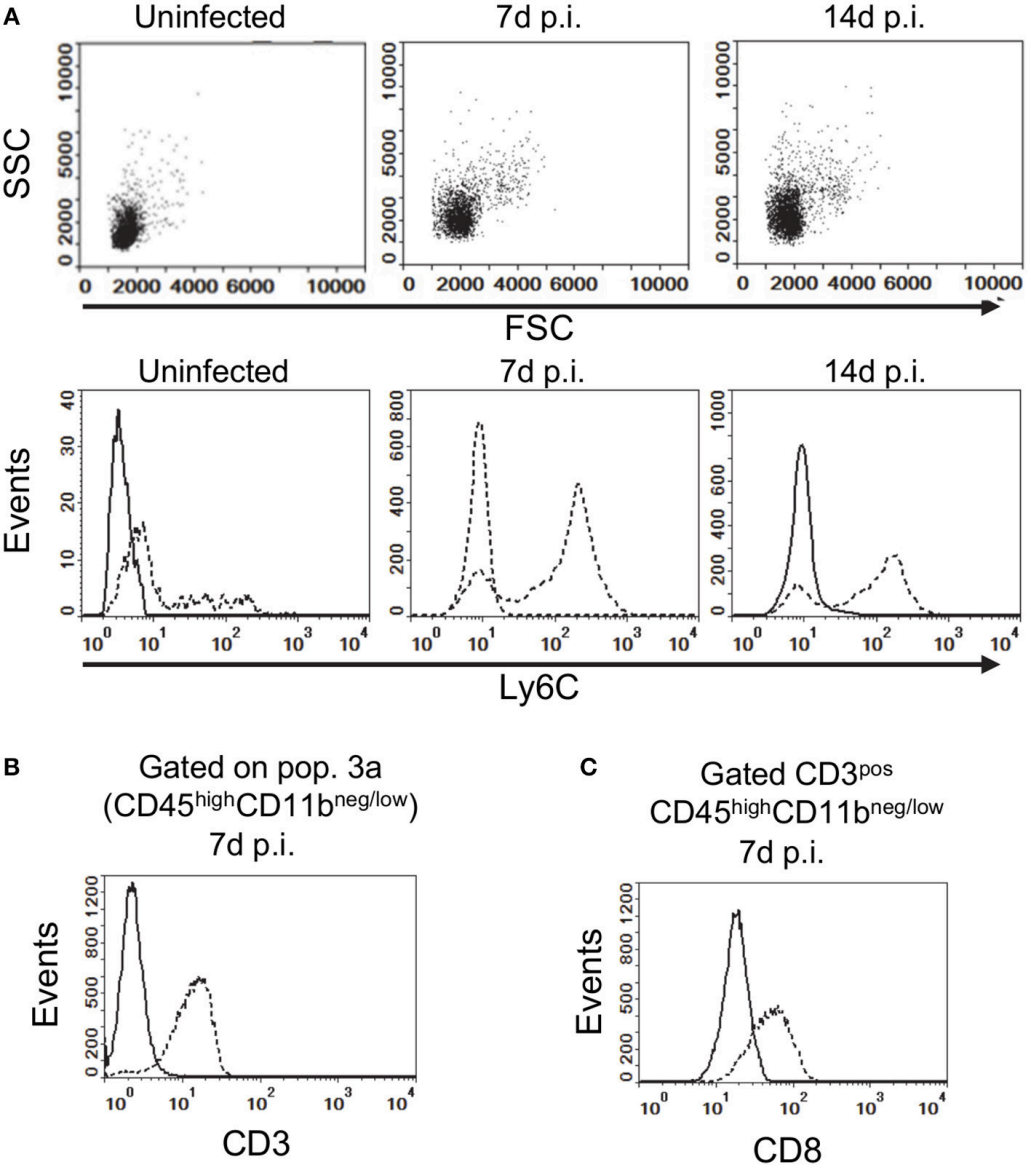
Gating strategy for analysis of brain leukocytes. **(A)** Gated CD45^high^CD11b^neg/low^ leukocytes from uninfected, and infected mice 7 d and 14 d p.i. projected as dotplots (upper panels) showing FCS vs. SSC, and histograms (lower panels) showing Ly6C expression. Histograms **(B,C)** show expressions of CD3 on CD45^high^CD11b^neg/low^ cells, and CD8a on gated CD3^pos^CD45^high^CD11b^neg/low^ cells (dotted lines), respectively, at 7 d p.i compared with isotype control (solid line). Representative plots are shown. Values given are gated events as a percentage of CD45^pos^ cells.

**Figure 3:**
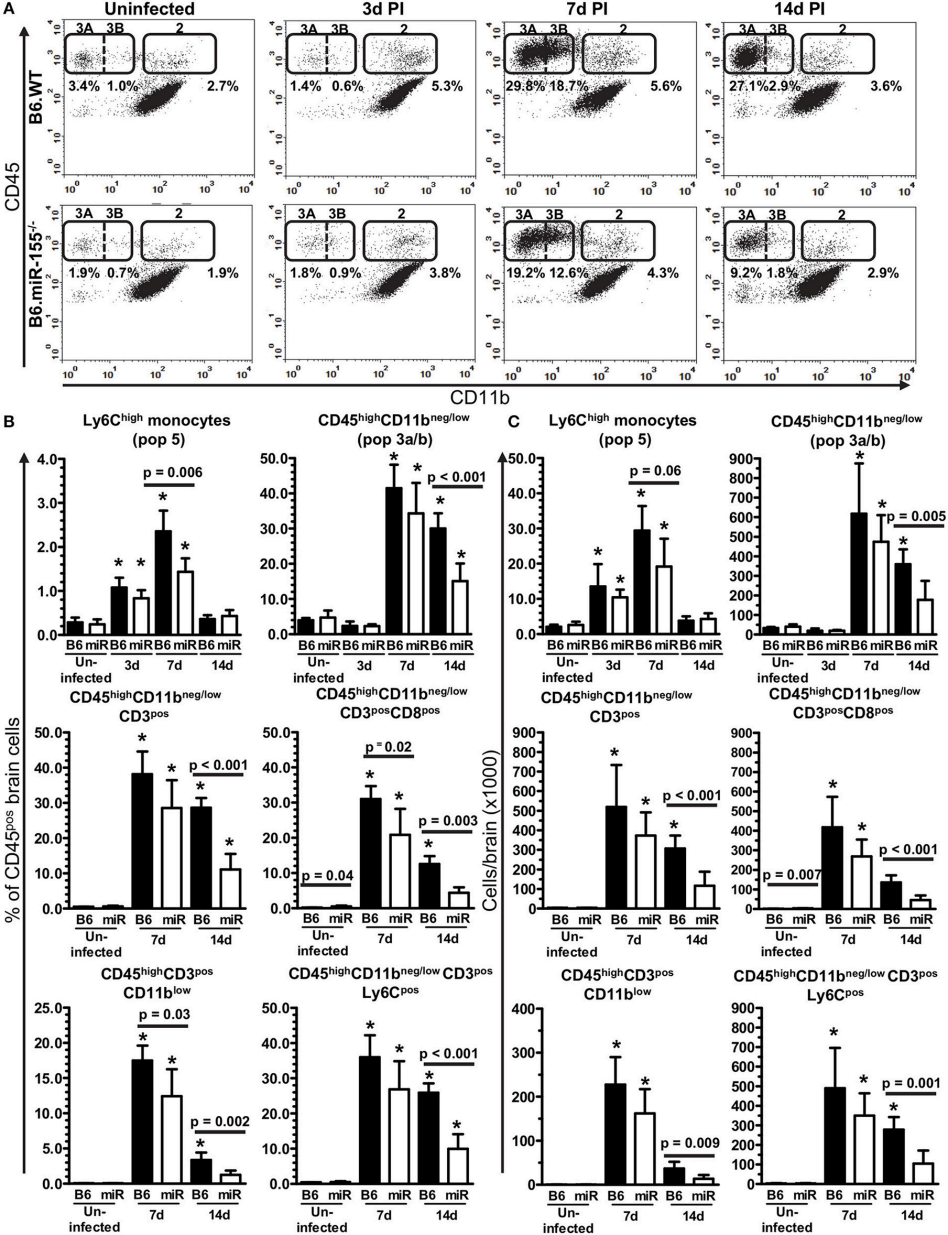
Lack of miR-155 impairs leukocyte recruitment to the brains of *L. monocytogenes* infected mice. Brain leukocytes were isolated from uninfected and infected age and sex-matched B6.WT and B6.miR-155^−/−^ mice described in Figure 2A at the indicated times and analyzed by flow cytometry. **(A)** CD45^pos^ cells were gated as in Figures 4A,B, dotplots from representative B6.WT (upper panels) and B6.miR-155^−/−^ (lower panels) animals are shown. Values given are gated events as a percentage of CD45^pos^ cells. Results in **(B,C)** are the mean + SD % CD45^pos^ cells **(B)**, or cells/brain **(C)** from 4–9 B6.WT (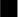) or B6.miR-155^−/−^ (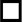) mice/time point. ^*^p < 0.05 compared with same genotype uninfected control by 1-way ANOVA with Dunnett's post-test. Significant *p*-values between genotypes calculated by 2-tailed Student's *t*-test are shown.

**Figure 4:**
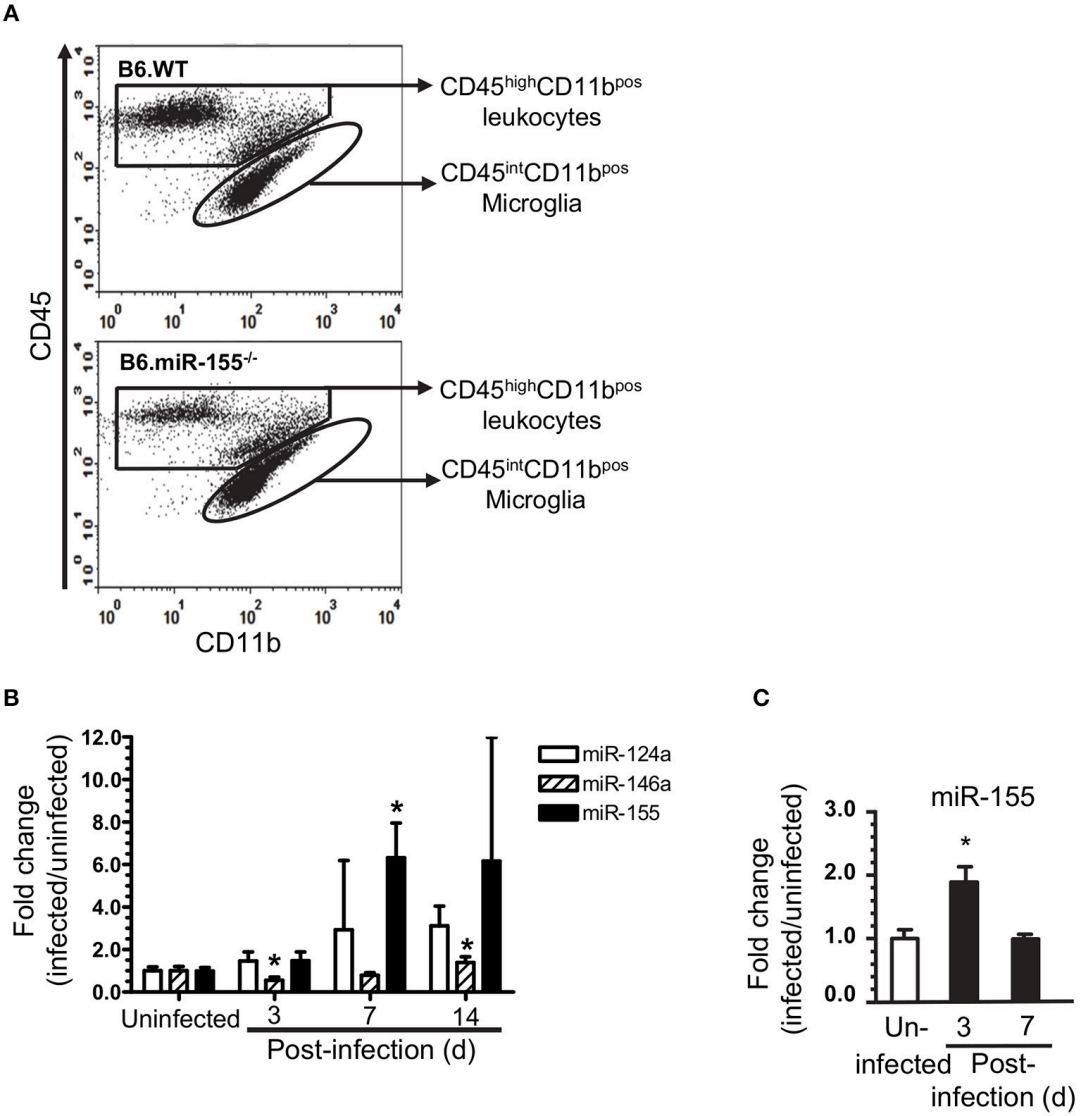
*L. monocytogenes* infection upregulates microglial miR-155. Brain leukocytes were isolated from uninfected and infected age and sex-matched B6.WT and B6.miR-155^−/−^ mice described in Figure 2A. Cells from 2 to 3 mice of each genotype per time point were pooled. CD11b^pos^ cells were collected from each cell pool by immunomagnetic sorting then underwent flow cytometric sorting to collect specific populations. Microglia, defined as CD45^int^CD11b^pos^ cells, were collected from uninfected mice, and mice 3, 7, and 14 d p.i. In contrast, CD45^high^CD11b^pos^ cells were collected at 7 d p.i. only. **(A)** Representative dot plots show sort windows 7 d p.i. **(B)** miR expression in sorted CD45^int^CD11b^pos^ microglia was measured by qPCR and normalized to snoRNA135. Results shown are fold change + SD miR expression (infected/uninfected) in 3–4 cell pools comprised of cells from 2 to 3 mice per time point. ^*^p < 0.05 compared with uninfected mice by one-way ANOVA with Dunnett's post-test. **(C)** miR-155 expression was determined by qPCR in CD11b^pos^ bone marrow cells and normalized to snoRNA. Results presented are the mean + SD fold change normalized miR-155 (infected/uninfected) from 4 individual mice of either genotype per time point. ^*^p < 0.05 compared with uninfected control by 1-way ANOVA with Dunnett's post-test.

**Figure 5:**
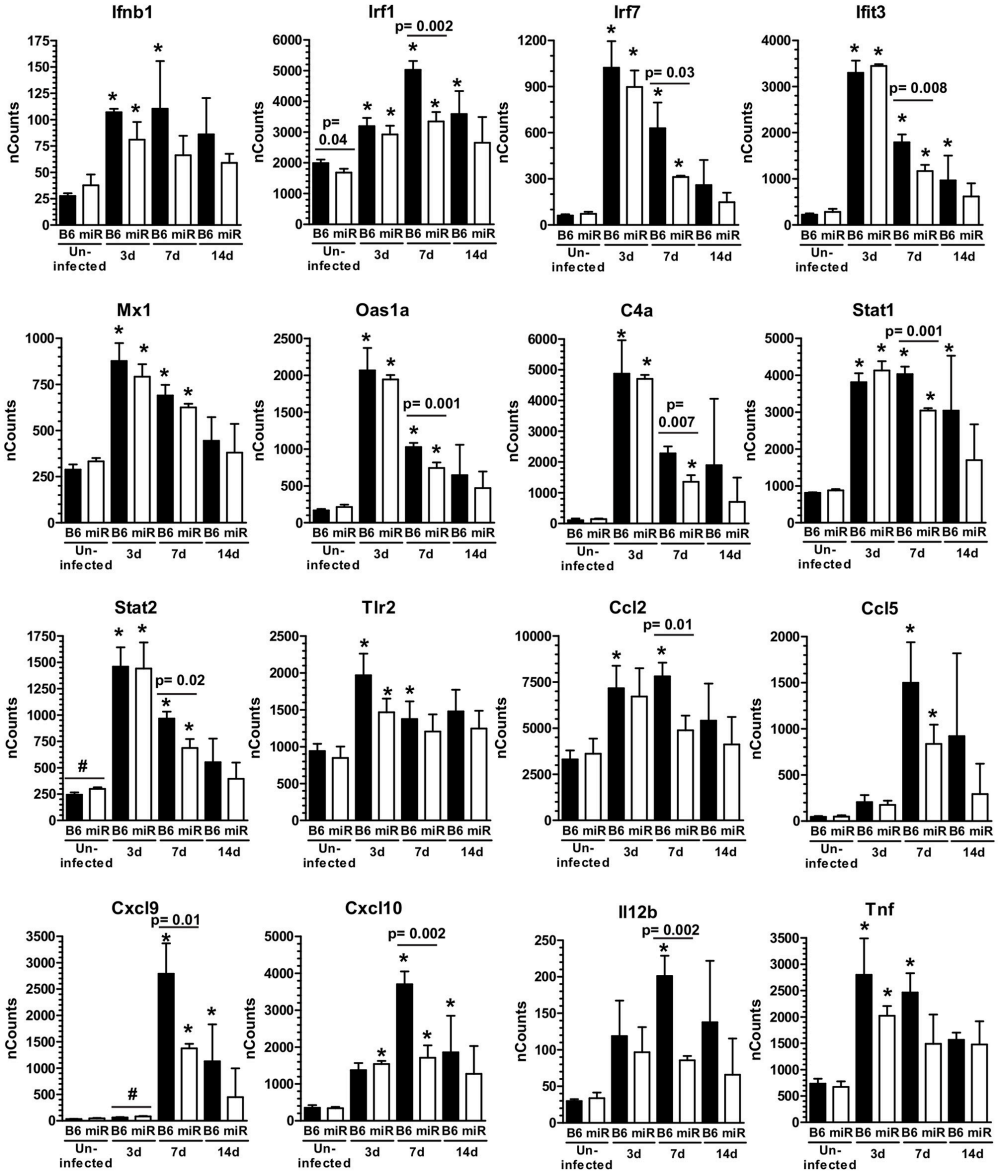
*L. monocytogenes* infection triggers IFN-activation of microglia. mRNA expression in microglia from *L. monocytogenes* EGD-infected male and female B6.WT (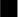) and B6.miR-155^−/−^ (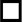) mice shown in Figure 3A was analyzed by nCounter. Results presented are the mean + SD nCounts from 3 pools of microglia per genotype at each time indicated. ^*^p < 0.05 compared with nCounts from uninfected mice of the same genotype by 1-way ANOVA with Dunnett's post-test. Significant *p*-values between genotypes calculated by 2-tailed Student's *t*-test are given.

**Figure 6:**
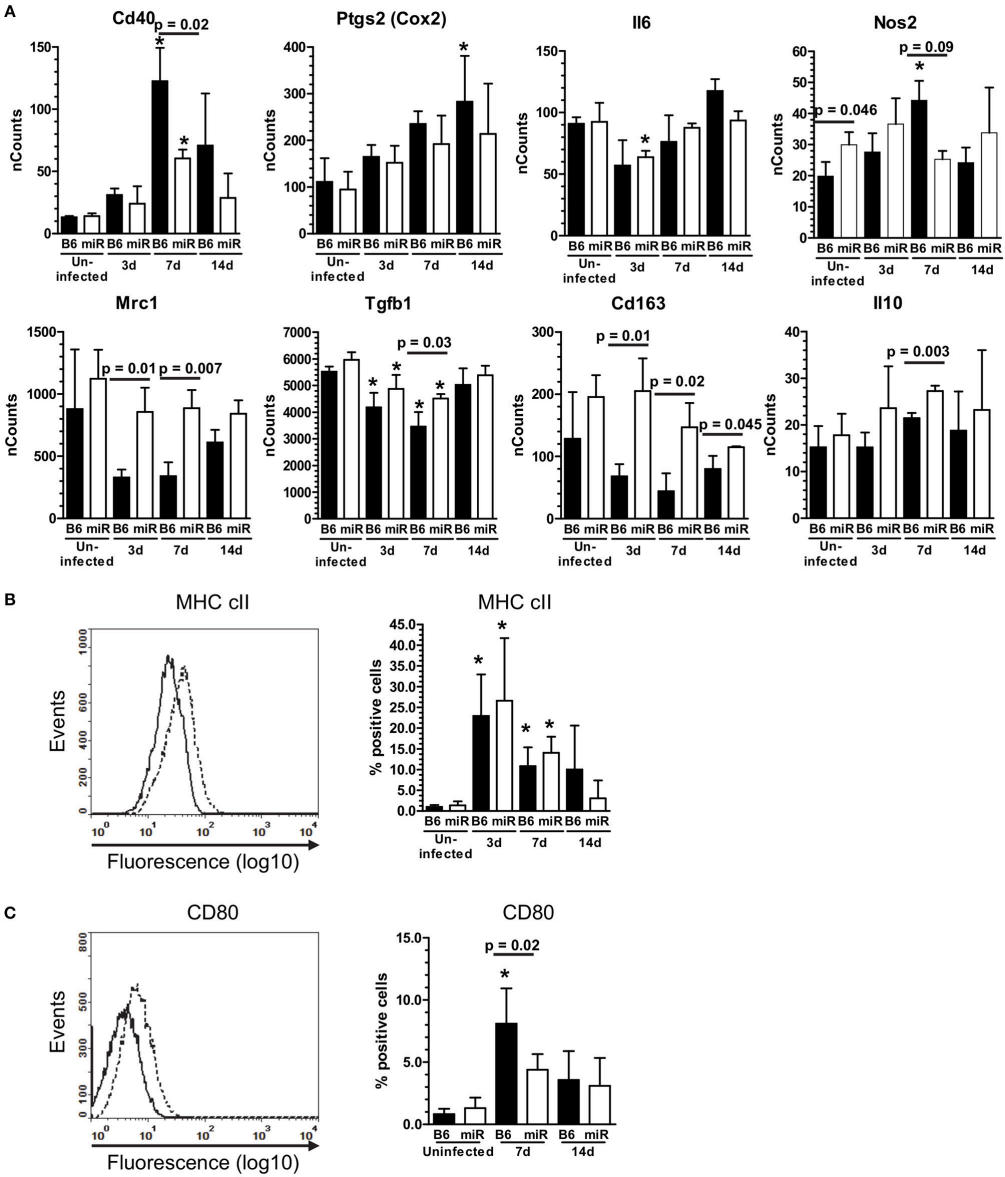
*L. monocytogenes* infection induces M1-polarization in microglia from C56BL/6 mice, but not B6.miR-155^−/−^ mice**. (A)** mRNA expression in microglia from *L. monocytogenes* EGD-infected male and female B6.WT (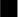) and B6.miR-155^−/−^ (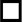) mice shown in Figure 5A was analyzed by nCounter. mRNA expression is presented as the mean ± SD nCounts from 3 pools of sorted microglia per genotype. **(B,C)** Brain cells from uninfected B6.WT and B6.miR-155^−/−^ mice and from mice infected with 3.3–4.2 × 10^5^ CFU *L. monocytogenes* EGD were incubated with the indicated mAb or isotype control mAb and analyzed by FACS. Microglia were gated as shown in Figure 4. Left panels show isotype (solid line) and test (dotted line) histograms from representative mice. Results from 3 experiments were combined and are presented are the mean + SD % marker positive microglia from B6.WT (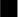) and B6.miR-155^−/−^ (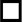) mice expressing MHC cII **(B)** or CD80 **(C)** at the indicated time (*n* = 4–17 mice/genotype). ^*^p < 0.05 compared with uninfected mice of the same genotype by 1-way ANOVA with Dunnett's post-test. Significant *p-*values between genotypes calculated by 2-tailed Student's *t*-test are given.

**Figure 7:**
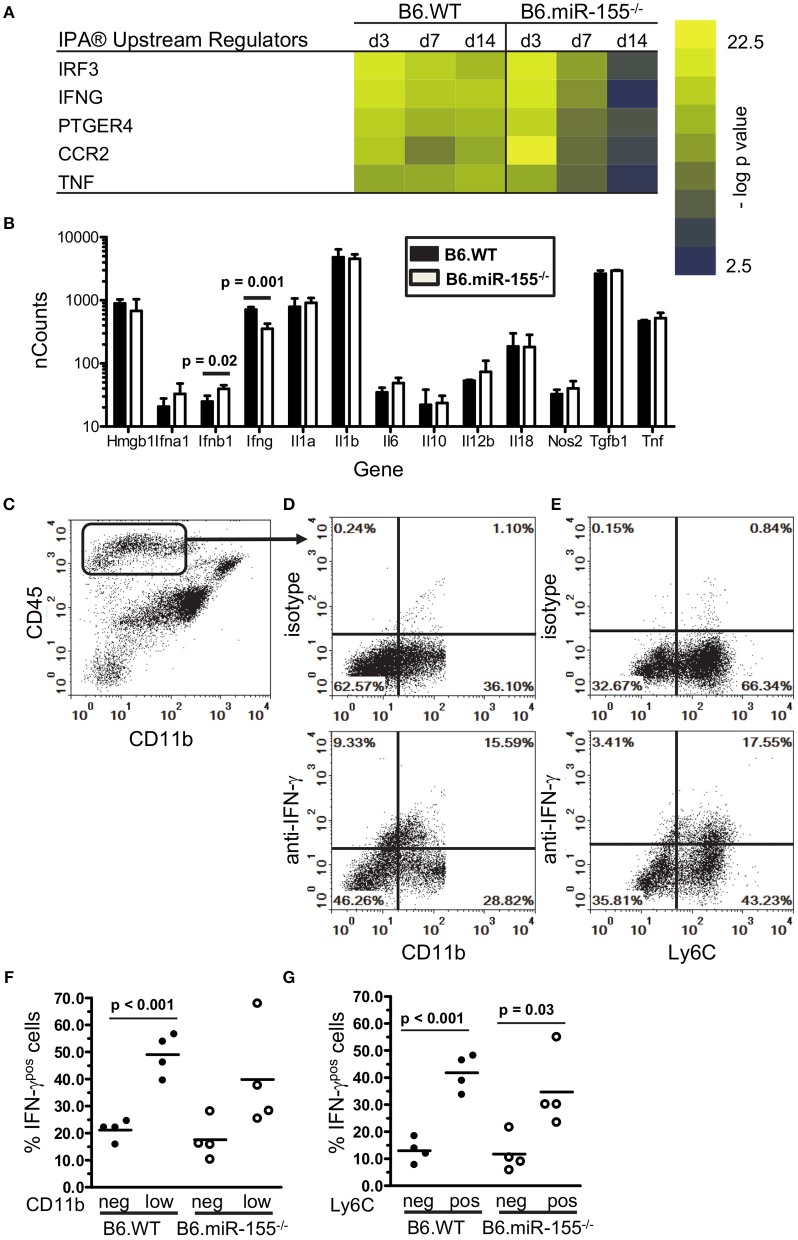
IFN-γ-producing CD45^high^ cells are recruited to brains of infected mice. **(A)** Heat map shows the top 5 upstream regulators of mRNA expression identified by IPA® Pathways Analysis in microglia from infected B6.WT and B6.miR-155^−/−^ mice. **(B)** CD45^high^CD11b^pos^ cells from brains of B6.WT (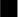) and B6.miR-155^−/−^ (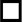) mice 7 d p.i. described in Figure 2 were collected by FACS sorting and mRNA transcripts were quantified by nCounter. Data presented are the mean + SD nCounts of the indicated transcripts from 3 to 4 cell pools, each derived from 2 to 3 mice/genotype. Significant *p*-values between genotypes calculated by 2-tailed Student's *t*-test are given. **(C–G)** Brain leukocytes were harvested at 7 d p.i. from B6.WT and B6.miR-155^−/−^ mice infected with 4.7 × 10^5^ CFU *L. monocytogenes* EGD then were incubated for 4 h with PMA/ionomycin plus brefeldin A and monensin. After immunolabeling of extracellular markers, the cells were fixed and permeabilized then incubated with anti-IFN-γ or isotype control mAb and analyzed by flow cytometry. **(C)** Dotplot shows gating of CD45^high^CD11b^neg/low^ cells from singlets. **(D,E)** Representative B6.WT dotplots show IFN-γ isotype (upper panels) and anti-IFN-γ (lower panels) mAbs vs. CD11b **(D)** and Ly6C **(E)** on gated CD45^high^CD11b^neg/low^ cells. Graphs **(F,G)** show percentages of IFN-γ^pos^ cells from B6.WT (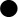) and B6.miR-155^−/−^ (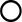) mice from 2 separate experiments that are grouped according to expression of CD11b **(F)** and Ly6C **(G)**. Line represents the group mean. Significant *p*-values calculated by 2-tailed Student's *t*-test are given.

This study was carried out in accordance with the recommendations of the Institutional Animal Care and use Committee (IACUC) of the University of Oklahoma HSC (OUHSC). All protocols were approved by the IACUC. All animals were purchased from Jackson Laboratories, Bar Harbor, ME. Female C57BL/6 (B6.WT) or pairs of aged matched, female B6.WT and B6.Cg-*Mir155*^*tm*1.1*Rsky*^/J (B6.miR-155^−/−^) mice were used in experiments depicted in Figures 1, **4C**, **7C,D**, **9A,B**, **10**, **12A–C** and Supplemental Figure 4. Age and sex-matched male and female B6.WT, or male and female B6.WT and B6.miR-155^−/−^ mice were used in experiments depicted in Figures 2, 3, 4A,B, 5, 6, 7A,B and Supplemental Figures 2A, 3. Animals were 10–20 weeks of age when used in experiments. Although the effects of different ages of mice were not studied, immunological differences in adult mice of this age, e.g., between 10 and 20 week old animals, are unlikely to have made a significant contribution (reviewed in (43). Mice were infected i.p. with 1 ml PBS containing 2–5 × 10^5^ CFU *L. monocytogenes* then were injected i.p. with 2 mg ampicillin (Butler Schein Animal Health, Dublin, OH) three times at 10–12 h intervals beginning 48 h post-infection (p.i.) (44). Bubblegum-flavored amoxicillin (2 mg/ml final concentration) was added to the drinking water 3 d p.i. and continued until 14 d p.i. Some uninfected mice received three doses of i.p. ampicillin, or three doses of i.p. ampicillin plus oral amoxicillin as described above to control for antibiotic effects. Mice were euthanized by CO_2_ asphyxiation, exsanguinated via femoral vein cut-down, and perfused trans-cardially with 25 ml iced, sterile PBS containing heparin 2 U/ml. Organs were removed aseptically and weighed, then were homogenized in dH_2_O for bacterial culture, placed in RNA*later* (ThermoFisher Scientific, Waltham, MA) for analysis of gene expression in whole organs, or were processed as described below for cellular analysis. Serial 10-fold dilutions were plated on tryptic soy agar and incubated at 37°C for 24 h and CFU bacteria were quantified.

### Tissue preparation and magnetic cell sorting

Perfused brains were digested using the Neural Tissue Dissociation T-kit, C-tubes, and a gentleMACS Dissociator according to manufacturer's protocol (Miltenyi Biotec, SanDiego, CA). Myelin was removed by centrifugation through 30% serum isotonic Percoll (Sigma-Aldrich, St. Louis, MO) 10 min at 700 × g at room temperature (RT)(45). Cells were collected and washed three times in PBS/0.5% BSA. Erythrocytes were lysed and cells were counted in a hemocytometer. CD11b^pos^ cells were isolated using CD11b Microglia Microbeads (Miltenyi Biotec, SanDiego, CA) and LS columns according to the manufacturer's protocol.

### SIM-A9 cells

The SIM-A9 mouse microglial cell line (ATCC, Rockville, MD) was cultured in DMEM/F12 medium containing 10% FBS and 5% horse serum. For infections with *L. monocytogenes*, 1 × 10^6^ SIM-A9 cells were co-cultured with *L. monocytogenes* in antibiotic free medium for 1 h, then were washed and incubated further in medium containing gentamicin (25 μg/ml). Cells were harvested 24 h after infection and expression of miR-155 was measured in triplicate samples of uninfected and infected cells by qPCR using total RNA isolated from cells. In other experiments, SIM-A9 were incubated 24 h with IFN-β (250 pg/ml) prior to measurement of gene expression. In addition, other IFN-β-treated cells were washed, then incubated another 24 h with IFN-γ (1.0 ng/ml), TNF (20 ng/ml), or IFN-γ (1.0 ng/ml) plus TNF (20 ng/ml) prior to analysis of gene expression by qPCR. Results were normalized to snoRNA135 for miR-155, or to *Gapdh* for *Ifit3* and *Cxcl9*. Fold changes were calculated against untreated cells.

For transfection, SIM-A9 cells were seeded in 6-well plates at a density of 5 × 10^5^ cells per well and incubated at 37°C, 5% CO_2_ with DMEM:F12 medium containing 5% horse serum and 10% FBS for 24 h. The following day, cells were transfected with 50 nM miRCURY LNA microRNA Inhibitor for mmu-miR-155*-*5p (miR-155 inhibitor) (Exiqon, Vedbaek, Denmark) or miRCURY LNA microRNA Inhibitor Control Negative Control A (scrambled control) (Exiqon, Vedbaek, Denmark) using Lipofectamine 2000 (ThermoFisher Scientific, Waltham, MA) in Opti-MEM according to manufacturer's protocol. After 6 h, the medium was replaced with DMEM:F12 containing 5% horse serum and 10% FBS for 18 h. Cells were then treated with 0.5, 1.0, and 2.0 ng/ml of IFN-γ for 24 h. qRT-PCR of miRNA was performed using TaqMan probes (ThermoFisher Scientific, Waltham, MA) for *mmu-*miR-155 to detect the transfection efficiency.

### Flow cytometry and multiplex ELISA

Cells were incubated 20 min on ice in PBS containing 0.5% BSA and 0.1% sodium azide with anti-CD16/32 mAb (BD Biosciences) prior to addition of isotype-matched control or test mAb, as previously described (46, 47). Cells were post-fixed by incubation in 4% formaldehyde for 20 min at room temperature prior to analysis. Flow cytometry was performed on a Stratedigm S1200Ex (Stratedigm Inc, San Jose, CA) and analyzed with CellCapTure software (Stratedigm Inc., San Jose, CA). To analyze leukocyte influxes during infection, CD45^pos^ cells were gated to show three main populations (Supplemental Figures 1A,B) including CD45^int^CD11b^pos^ microglia (Pop. 1), CD45^high^CD11b^high^ leukocytes (Pop. 2) and CD45^high^CD11b^neg/low^ leukocytes (Pop 3). CD45^high^CD11b^high^ leukocytes were sub-divided into Ly6G^pos^ neutrophils (Pop 4), and Ly6C^high^ monocytes (Pop 5), and CD45^high^CD11b^neg/low^ leukocytes were sub-divided into CD11b^neg^ (Pop. 3a) and CD11b^low^ (Pop. 3b) groups.

For measurement of cytokine production, brain cells (2.5 × 10^5^/ml) were incubated overnight in DMEM/F12 medium containing 10% FCS plus penicillin/streptomycin with heat-killed (HK) *L. monocytogenes* as described (48). Brefeldin A (eBioscience) was added for the last 4–6 h of incubation. Supernatants were collected and stored at −80°C. Cells were collected and incubated with mAb against CD11b, CD45, Ly6C, and Ly6G then were fixed and permeabilized using intracellular fixation and permeabilization buffers (eBioscience, San Diego, CA) according to the manufacturer's protocol. Subsequently, cells were incubated with anti-IFN-γ or with fluorochrome- and isotype-matched control mAb then were analyzed by flow cytometry. Cytokine expression in specific cell populations was determined by subtracting the % of positive cells obtained with control antibodies from the % of positive cells obtained using test antibodies.

Concentrations of specific cytokines in cell supernatents were measured on a Bio-Plex 200 using Bio-Plex Pro magnetic beads (Bio-Rad, Hercules, CA) according to the manufactorers protocol. When results for a given group included cytokine target concentrations below the limit of detection, these values were set at 50% below the lowest standard for calculating the group mean.

### Nanostring cell preparation and analysis

CD11b^pos^ brain cells were collected from uninfected mice and from mice 3, 7, and 14 dPI. Cells from 2 to 3 mice per group were pooled and incubated with mAb against CD45 and CD11b (clone 5C6) and analyzed on a FACSJazz (BD Biosciences) cell sorter. Microglia, identified as CD45^int^CD11b^pos^ cells, were collected at each time point. In addition, at 7dPI CD45^high^CD11b^pos^ cells were collected due to their abundance at this time point. Total RNA from the sorted cells was extracted using miRNeasy Micro Kit from Qiagen (Redwood City, CA) for purification of total RNA from small amounts of cells according to the manufacturer's instructions. mRNA expression analysis with the NanoString nCounter® Mouse Inflammation v2 Panel was performed according to manufacturer's protocol (NanoString Technologies Inc., Seattle, WA). nCounts were normalized to the geometric mean of the internal positive controls using nSolver™ Analysis Software (NanoString Technologies Inc., Seattle, WA). Results shown are the mean ± SD nCounts from 3 to 4 cell pool/time point. Statistical analysis of nCounts within the same genotype across was performed by 1-way ANOVA. Post-test alpha-level adjustment was accomplished by Dunnett's method or in some cases Tukey's Multiple Comparison Test. Comparisons between genotypes were performed using two-tailed Student's *t*-test with equal variance. In addition, log-transformed data from nSolver were directly uploaded into Ingenuity® Pathway Analysis software (Qiagen, Redwood City, CA) for analysis of Upstream Regulators. Assessment of gene regulation by Types I, II, and III IFNs was determined using the Interferome 2.0 database (49).

### Quantitative real-time PCR

RNA was extracted from mouse brains using a standard protocol applying the direct-zol RNA Miniprep kit from Zymo Research (Irvine, CA) according to the manufacturer's instructions. Total RNA was reverse transcribed using iScript™ cDNA Synthesis Kit (BioRad, Hercules, CA). Real time PCR reactions were run with SYBR® Green PCR master mix (Applied Biosystems, Foster City, CA) at 20 μl volumes in 96-well optical reaction plates using the BioRad C1000 Thermal Cycler and CFX96 Real-Time systems. Primers used for mRNA targets for *Fos, Ifng, Mapk1, Socs1, Tab2, Tnf* were obtained from PrimerBank (https://pga.mgh.harvard.edu/primerbank), *Gapdh* was previously published (50), and *Il1b* was designed using Beacon Designer 4 (Premier Biosoft International, Palo Alto, CA). Sequences are listed in Supplemental Table 1. Thermal-cycling conditions were as follows: hold at 95°C for 10 min, 40 cycles of 95°C for 15 s and 60°C for 1 min, hold at 95°C for 5 s, hold at 65°C for 5 s, ramp to 95°C with increments of 0.5°C. Melting curve analysis was performed, and no formation of primer-dimers or non-specific amplification products were identified. The ddCt method (51) was used to estimate relative changes in mRNA expression using *Gapdh* as housekeeping gene. The results are expressed as fold changes relative to control group. To determine miRNA expression, TaqMan microRNA reverse transcription kit and TaqMan miRNA assay kit from Life Technologies were used following the manufacturer's protocol. Primers for miR-155 (assay ID 002571) miR-124a (assay ID 001182), miR-146a (assay ID 000468) and snoRNA 135 (assay ID 001230) were purchased from Invitrogen (Carlsbad, CA) and used in standard TaqMan assays. The expression of miRNA was normalized to snoRNA-135.

### Statistical analysis

Data were analyzed using two-tailed Student's *t*-test with equal variance for experiments with two groups, or one-way ANOVA for experiments with multiple groups followed by a *post-hoc* comparison, using Dunnett's method for alpha-level adjustment, if the initial, overall test showed a *p* < 0.05. For skewed distributions, the Kruskal–Wallis test with Dunn's post-test was used to compare median values among three or more experimental groups. Modeling assumptions for parametric methods were evaluated and when assumptions were not met, as in the case of skewed distributions, a non-parametric method, the Kruskal–Wallis test, was used. Statistical significance between test and control groups was set with a *p* < 0.05. To assess differential susceptibility to infection, the time to early death distributions were estimated using the Kaplan–Meier method and were compared between the normal and B6.miR-155^−/−^ mice using a log-rank test. Kaplan–Meier-based estimated percentages for early death are greater than those calculated using the total number of mice in the denominator because the Kaplan–Meier method accounts for the differential follow-up of the mice with targeted harvest dates ranging from 3 to 14 days.

## Results

### *L. monocytogenes* infection upregulates brain miR-155

The goal of these studies was to analyze miR-155 expression and function during neuroinvasive bacterial infection and its treatment with antibiotics. For this we modified the mouse model of experimental listeriosis such that B6.WT mice received a lethal inoculum of *L. monocytogenes* EGD i.p, then were treated with antibiotics to kill *L. monocytogenes* starting 48 h post-infection (p.i.) (Figure 1A). Preliminary studies confirmed brain infection by culturing bacteria from 5 of 8 mice at 72 h PI, i.e., 24 h after starting antibiotics, with a mean (± SD) of 242 ± 302 CFU *L. monocytogenes*/brain (range 32–720). Analysis of gene expression in whole brain confirmed infection triggered CNS inflammation by showing upregulation of mRNA for IL-1β, TNF, and IFN-γ (Figure 1A). There was a significant increase in miR-155 expression at 7 d p.i., but it was not upregulated in brains of uninfected antibiotic-treated mice (Figure 1B). In contrast, expressions of miR-124a and miR-146a were unchanged. These experiments show antibiotic rescue of mice after lethal, systemic *L. monocytogenes* infection can be used to study inflammatory responses in the brain in surviving animals. Initial results show infection upregulates gene expression for key pro-inflammatory cytokines in the brain. In addition, miR-155 is also upregulated suggesting that it could play a role in brain inflammation in this model.

### *L. monocytogenes* infection of B6.miR-155^−/−^ mice

To understand more completely the contribution of miR-155 to brain inflammation during severe *L. monocytogenes* infection, age, and sex matched B6.WTand B6.miR-155^−/−^ mice were infected with a lethal inoculum of *L. monocytogenes* EGD (3.4 ± 0.33 × 10^5^ CFU/mouse) then treated with antibiotics as described above. In these experiments differential survival between the genotypes was not observed. Death occurred between days 2 and 7 p.i. with an estimated cumulative incidence of early death before day 14 of 8.3% (95% confidence interval: 3.1% to 20.2%) among B6.WT mice and 12.6% (95% CI: 5.8–25.4%) among B6.miR-155^−/−^ mice, (log-rank test: *p* = 0.52) (Supplemental Figure 2A). Differences between male and female mice in susceptibility to *L. monocytogenes* infection have been reported by some (52) but not others (53, 54). In our experiments, differences in mortality between male and female mice were not observed. Among 25 male and 25 female B6.miR-155^−/−^, 3 male, and 3 female mice succumbed to infection as did 1 male and 3 female among 25 male and 25 female B6.WT mice (log-rank test: *p* = 0.47). Microbiological analysis at 3 d p.i. showed the median bacterial load in the liver was 5.5-fold lower (*p* < 0.05) in female B6.miR-155^−/−^ mice than in B6.WT mice, but bacterial loads in spleen were not different between the genotypes (Supplemental Figure 2B). Bacterial loads in male mice were not collected. As expected given the effect of antibiotics, all spleens and livers were sterile at D7PI (*n* = 9–12/genotype). Brain infection was not different between the genotypes, with bacteria detected in 2 of 8 B6.WT mice and 1 of 8 B6.miR-155^−/−^ mice at 3 d p.i., and in 1 of 6 B6.WT mice and 2 of 8 B6.miR-155^−/−^ mice at 6 d PI.

Flow cytometry was used to assess infiltration of the brain by CD45^high^ bone marrow-derived leukocytes during infection. CD45^high^CD11b^neg/low^ leukocytes (Pop 3) showed infection-induced changes included increased forward and side scatter characteristics and greater Ly6C expression (Figure 2A). Preliminary analysis of lymphocyte markers showed CD45^high^CD11b^neg/low^ cells expressed CD3 and that CD3^pos^CD45^high^CD11b^neg/low^ cells also expressed CD8a (Figures 2B,C). CD4 was not consistently detected due to technical issues although CD4^pos^ T-lymphocytes are likely present (44). These results suggest the CD45^high^CD11b^neg/low^ population contains CD3^pos^-T-lymphocytes recruited into the brain during the primary immune response to *L. monocytogenes* (55–58).

Figure 3A shows representative dotplots in uninfected and infected mice of both genotypes illustrating CD45^high^ leukocytes entering the brain during infection. There were significant increases of Ly6C^high^ monocytes at 3 and 7 d p.i. in both genotypes (Figures 3B,C). Results at 7 d p.i. suggested significantly greater accumulation of these cells in B6.WT mice than in B6.miR-155^−/−^ mice measured as a percentage of CD45^pos^ cells, whereas differences in numbers showed a strong trend (*p* = 0.06). Numbers of neutrophils also increased significantly in B6.WT mice at 7 d p.i. from a mean (±SD) of 8,429 ± 2,532 (*n* = 9) in uninfected mice to 17,275 ± 2,906 (*n* = 7) at 7 d p.i. (*p* < 0.001 by one way ANOVA with Dunnett's post-test). However, numbers of neutrophils did not change significantly in B6.miR-155^−/−^ mice and differences between the genotypes were not significant (**data not shown**). Compared with Ly6C^high^ monocytes and neutrophils, many more CD45^high^CD11b^neg/low^ cells (Pop. 3a/b) entered the brains of both genotypes at 7 d p.i. with significantly fewer of these cells in B6.miR-155^−/−^ mice at 14 d p.i. (Figures 3B,C). Within the CD45^high^CD11b^neg/low^ population, B6.miR-155^−/−^ mice had significantly fewer CD3^pos^ and CD3^pos^ CD8^pos^ T-lymphocytes at 14 d p.i. than did B6.WT mice. Additionally, CD3^pos^CD8^pos^ cells were also reduced at 7 d p.i. in B6.miR-155^−/−^ mice when measured as a percentage of CD45^pos^ cells. Analysis of CD11b and Ly6C expression on CD3^pos^ T-lymphocytes in the CD45^high^CD11b^neg/low^ population as surrogate activation markers also showed activated cells were significantly lower in B6.miR-155^−/−^ mice than in B6.WT mice at 14 d p.i. Collectively, these data show that miR-155 promotes recruitment of inflammatory cells, in particular activated T-lymphocytes, to the brain during neuroinvasive *L. monocytogenes* infection.

### Microglial gene expression during *L*. *monocytogenes* infection

To understand the role of microglial miR-155 during neuroinvasive infection more precisely, we measured miR expression and compared gene expression signatures in microglia isolated by flow cytometric sorting from *L. monocytogenes* EGD*-*infected B6.WT and B6.miR-155^−/−^ mice (Figure 4A). Similar to prior results in whole brain and CD11b^pos^ cells, miR-155 expression was unchanged at 3 d p.i., but was upregulated 7 d p.i. (Figure 4B). Although miR-155 expression was >2.5-fold higher in each of 4 samples from mice 14 d p.i. compared with uninfected mice, this did not achieve statistical significance. Expression of miR-124a did not change significantly, whereas miR-146a expression decreased at 3 d p.i. and increased by 14 d p.i., albeit with lesser magnitude than miR-155^−/−^. By comparison, miR-155 was upregulated at 3 d p.i. in CD11b^pos^ bone marrow cells but returned to baseline by d 7 p.i. (Figure 4C). These data confirm systemic infection with fully virulent *L. monocytogenes* upregulates miR-155 in microglia, with a delayed kinetic compared to its upregulation in bone marrow CD11b^pos^ cells.

Analysis of gene expression showed significant upregulation of IFN-associated genes, including transcription factors, antiviral genes, complement components, and downstream pro-inflammatory mediators in microglia of both genotypes at 3 d p.i. (Figure 5). Upregulation of *Ifit3* and *Oas1* show a key role for stimulation by Type I IFN (59). Numbers of expressed transcripts were largely similar in B6.WT and B6.miR-155^−/−^ mice at 3 d p.i. However, by 7 d p.i. nCounts of several IFN-associated genes were significantly higher in B6.WT mice than in B6.miR-155^−/−^ mice. The magnitude of gene expression was declining by 14 d p.i., but expression of IFN-related genes including *Irf7, Ifit3, Stat1, Cxcl9*, and *Cxcl10* remained elevated in B6.WT mice over uninfected controls (Figure 5). In contrast, their expression in B6.miR-155^−/−^ mice at 14 d p.i. was not different from uninfected B6.miR-155^−/−^ mice. Additionally, upregulation of genes such as *Ccl5, Cd40, Cxcl10, Il12b, Nos2, Tnf*, and *Ptgs2* (*Cox2*) indicated polarization along a classical/M1 pathway in B6.WT mice (60) (Figures 5, 6A). In contrast, markers of M1 polarization were not expressed to the same degree in B6.miR-155^−/−^ mice, or were not significantly upregulated in them.

Markers of M2a and M2b/c polarization including *Mrc1 (Cd206), Tgfb1, Arg1, Cd163, Chil3l3* (*Ym1*), *Retnl* (*Fizz1*), and *Il10* were not upregulated in either genotype (Figure 6A and Supplemental Data nCounts). However, expressions of *Mrc1, Tgfb1, Cd163*, and *Il10* were greater in cells from B6.miR-155^−/−^ mice. With the exception of *Il10*, expression of these markers was reduced to a greater extent in B6.WT mice than in B6.miR-155^−/−^ mice. Analysis of surface markers by flow cytometry showed significant upregulation of MHCcII on microglia from both genotypes, and of CD80 on cells from B6.WT mice (Figures 6B,C). These data show microglia in B6.WT mice develop an IFN-activation signature by d 3 p.i. and express key features of M1 polarization at 7 d p.i. The signature waned by 14 d p.i but was still evident. Markers of IFN-activation in B6.miR-155^−/−^ mice are quite similar to B6.WT mice at 3 d p.i. However, B6.miR-155^−/−^ microglia do not achieve the same degree of genotypic polarization and features of IFN activation return to baseline more rapidly.

### Reduced IFN-γ activation of microglia in B6.miR-155^−/−^ mice

Interestingly, expressions of some IFN-related genes increased significantly between 3 and 7 d p.i. in B6.WT mice (*Cxcl9 p* < 0.001, *Cxcl10 p* < 0.001*, Irf1 p* < 0.01 & *Cd40 p* < 0.01) and also in B6.miR155^−/−^ mice (*Cxcl9 p* < 0.01, *Cd40 p* < 0.05 by Tukey's Multiple Comparison Test) (Figure 5). We hypothesized expression of these genes was stimulated by cytokines produced by bone marrow-derived cells that had entered the brain by 7 d p.i. (61). Indeed, IPA® upstream regulators of microglial responses showed prominent roles for IFN-γ and TNF (Figure 7A), and high transcript numbers of both cytokines, were found in CD45^high^CD11b^pos^ brains cells at 7 d p.i. (Figure 7B). Notably, there were 2-fold fewer *Ifng* transcripts in cells from B6.miR-155^−/−^ mice than in cells from B6.WT mice. In contrast, there were no differences between the genotypes in expression of other mediators e.g., *Tnf* , *Il1a*, and *Il1b*, the exception being *Ifnb*, which was expressed at levels < 10-fold lower than *Ifng*. Flow cytometric analysis of the CD45^high^CD11b^pos^ cells used for gene expression analysis (Figure 4A) showed samples from B6.miR-155^−/−^ mice contained lower percentages and numbers of CD45^high^CD11b^low^ cells in compared with samples from B6.WT mice. This sub-population comprised 17.1 ± 1.3% (*n* = 4 cell pools) of CD45^high^ cells compared to 30.0 ± 5.5% (*n* = 3 cell pool, *p* = 0.01), and contained 40,580 ± 6860 cells compared to 98,813 ± 12,664 cells (*p* = 0.002) in B6.miR-155^−/−^ and B6.WT mice, respectively. These results indicate that bone-marrow derived cells are a source of cytokines that can stimulate microglia. In addition, they suggest microglia from B6.miR-155^−/−^ mice could be exposed to less IFN-γ stimulation *in vivo* than microglia from B6.WT mice.

To identify the main source of IFN-γ, CD45^high^CD11b^pos^ brain cells were collected 7 d p.i. and stimulated with PMA/ionomycin. Cells that produced IFN-γ were quantified by flow cytometry (Figures 7C–G). Similar percentages of cells from each genotype were IFN-γ^pos^ by this method. In B6.WT mice, 28.7% ± 3.6 (mean ± SD, *n* = 4) of CD45^high^CD11b^neg/low^ cells were IFN-γ^pos^, and in B6.miR-155^−/−^ mice, 27.3% ± 11.1 of cells were IFN-γ^pos^. Next, we analyzed IFN-γ expression in sub-populations of CD45^high^CD11b^neg/low^ brain cells according to low and negative expression of CD11b (Figures 7D,F), and to negative and positive expression of Ly6C (Figures 7E,G). This showed more IFN-γ^pos^ cells among the CD11b^low^ cells than among the CD11b^neg^ cells in B6.WT mice (Figure 7F). In addition, higher IFN-γ production was found among Ly6C-expressing cells in both genotypes (Figure 7G). IFN-γ^pos^ cells were also found among monocytes (CD45^high^CD11b^pos^Ly6C^high^), but at a much lower frequency than among the CD45^high^CD11b^neg/lo^ brain cells and with no difference between genotypes. Collectively, these data support the idea that influxes of activated lymphocytes entering the CNS are a major source of IFN-γ that could provide a second stimulus to microglia activated during the innate immune response. Unfortunately, the non-specific stimulus provided by PMA was not able to identify differential IFN-γ production between the genotypes, which requires an antigen-specific stimulus (34, 35).

**Figure 8:**
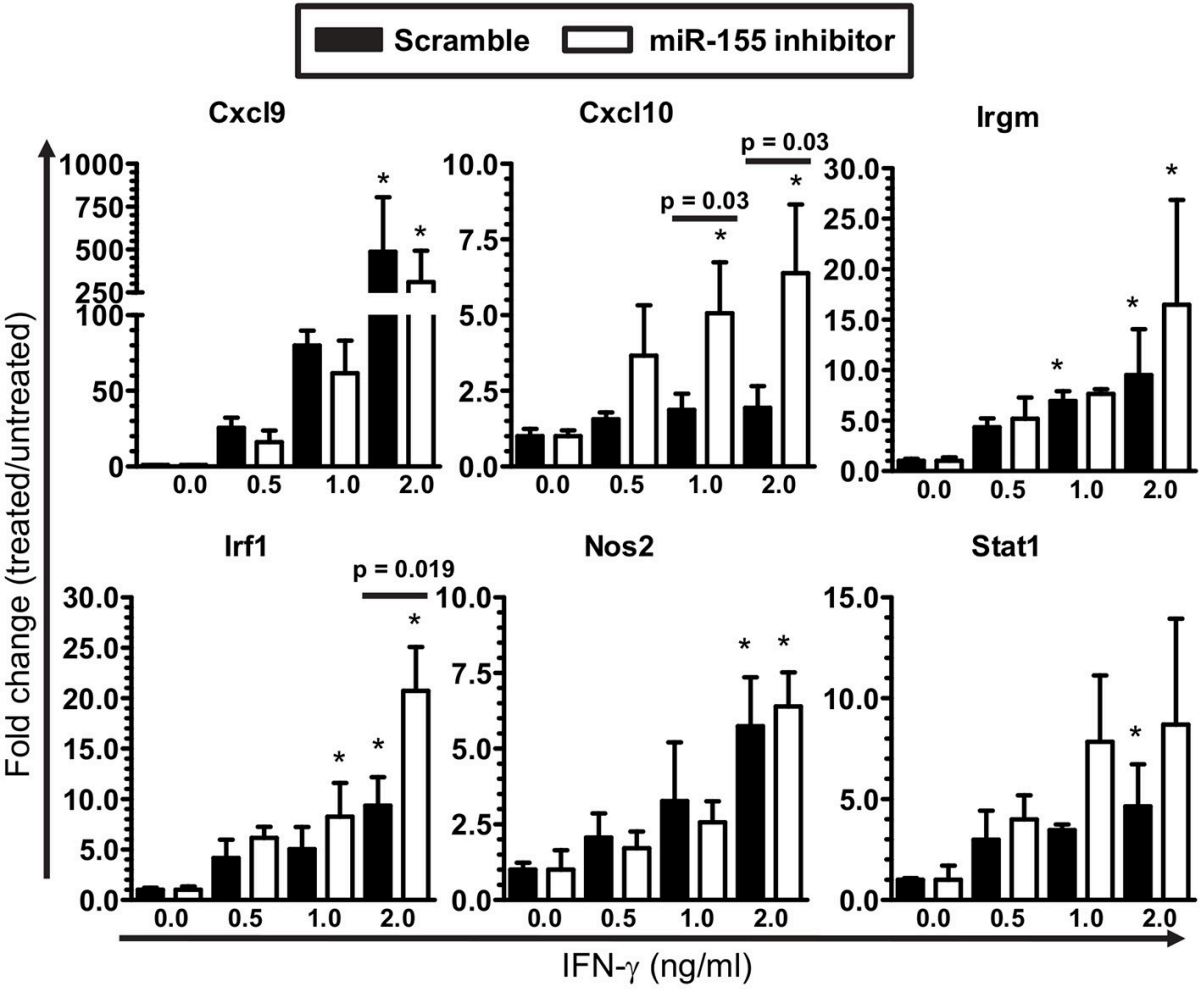
IFN-γ activation of microglia is not limited by miR-155 knock-down. SIM-A9 microglial cells were transfected with 50 nM inhibitor for mmu-miR-155-5p (miR-155 inhibitor) or control (scramble) then were incubated with increasing concentrations of IFN-γ for 24 h. Expression of the indicated genes was measured by qPCR and normalized to *Gapdh*. ^*^p < 0.05 compared with untreated cells transfected with scramble or miR-155 inhibitor determined by 1-way ANOVA with Dunnett's post-test. Significant *p*-values between inhibitor and scramble groups treated with the same concentration of IFN-γ were calculated by 2-tailed Student's *t*-test and are given.

The finding of fewer *Ifng* transcripts in CD45^high^CD11b^neg/low^ cells from B6.miR-155^−/−^ mice (Figure 7B) suggested deficient IFN-γ stimulation could account for lower nCounts of IFN-related genes e.g., *Cxcl9, Cxcl0, Irf1*, and *Stat1* in their microglia at 7 d p.i. (Figure 5). This was tested in SIM-A9 microglial cells transfected with miR-155 inhibitor or control (scramble), followed by IFN-γ stimulation (Figure 8). Indeed, miR-155 knock-down did not impair expression of IFN-γ-induced genes including *Cxcl9, Cxcl10, Irf1, Irgm1, Nos2*, and *Stat1*. These data suggest lower expression of IFN-induced genes observed in B6.miR-155^−/−^ microglia at 7 d p.i. resulted from reduced IFN-γ stimulation rather than an impaired ability to respond to the stimulus.

### Investigation of factors that upregulate brain miR-155 *in vivo*

**Figure 9:**
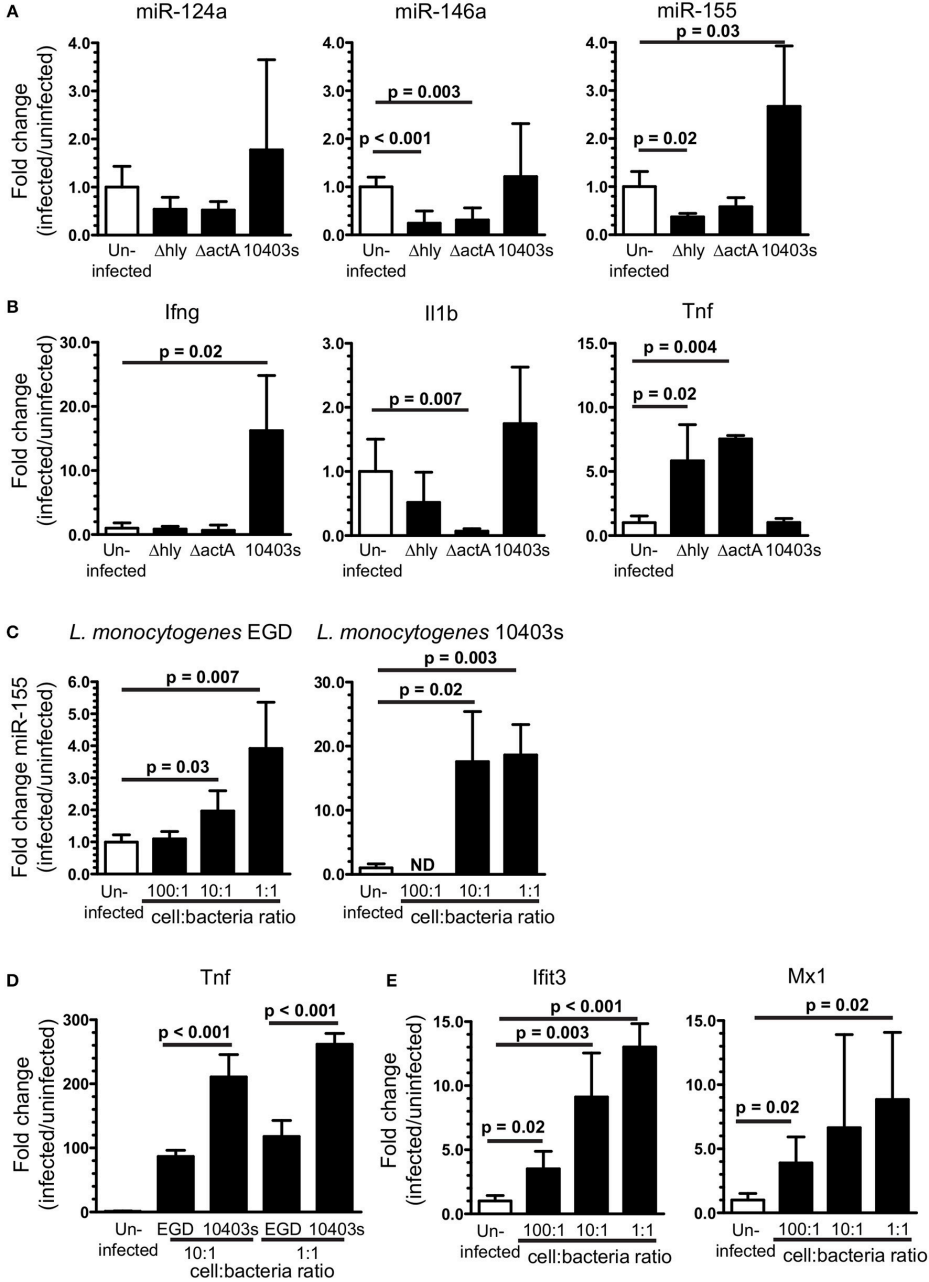
Upregulation of brain miR-155 *in vivo* requires infection with neuroinvasive bacteria. B6.WT mice were infected with *L. monocytogenes* strain 10403s (7 × 10^5^ CFU), or with non-neuroinvasive 10403s-derived Δ*hly* and Δ*actA* mutants (2 × 10^7^ CFU), then were treated with antibiotics. Brains were harvested 7 d p.i. and expressions of miR **(A)** and mRNA **(B)** were measured by qPCR and normalized to snoRNA135 and *Gapdh*, respectively. Results presented are the mean + SD fold change (infected/uninfected) normalized gene expression from 3 to 6 mice/genotype at each time point. **(C,D)** 10^6^ SIM-A9 cells were incubated with increasing numbers of *L. monocytogenes* EGD or 10403s. Gentamicin (25 μg/ml) was added after 1 h and the cells were harvested 24 h after infection. Expressions of miR-155 **(C)**, *Tnf* **(D)**, and *Ifit3* and *Mx1* **(E)** were measured by qPCR and normalized to snoRNA135 **(C)** or *Gapdh* **(D,E)**. Expressions of *Ifit3* and *Mx1* were measured only in cells infected with EGD. Fold changes were calculated against uninfected cells. Significant *p*-values between groups calculated by 2-tailed Student's *t*-test or Mann–Whitney *U*-test are given. ND, not done.

Results above show microglia are stimulated by Type I IFNs produced during the innate immune response to *L. monocytogenes* infection. Intracellular infection of macrophages also triggers expression of IFN-β and downstream genes and can upregulate miR-155 *in vitro* (62, 63). Thus, we pursued experiments testing bacterial roles of neuroinvasion and intracellular infection of microglia on miR-155 upregulation and activation in this model. Mice were infected with virulent *L. monocytogenes* strain 10403s (7.0 × 10^5^ CFU/mouse) or with avirulent *L. monocytogenes* 10403s-derived Δ*hly* and Δ*actA* mutants (2.0 × 10^7^ CFU/mouse), neither of which invades the CNS (44, 46). Notably, Δ*actA* mutants escape phagosomes, spread cell-to-cell, and activate cytosolic surveillance mechanisms and IFN-β production, whereas Δ*hly* mutants are retained in phagosomes and killed [reviewed in (64)]. Results in Figure 9A show 10403s, but neither mutant, upregulated miR-155 in the brain at 7 d p.i. Interestingly, strain 10403s also stimulated *Ifng* upregulation (Figure 9B) as did strain EGD (Figure 1A). In contrast to strain EGD, neither 10403s nor its mutants upregulated *Il1b* whereas both mutants, but not the parent strain, upregulated *Tnf* (Figure 9B). These results suggest neuroinvasion is required for upregulating miR-155 *in vivo*. Although between strains EGD and 10403s may be reflected in different CNS responses (65), induction of IFN-γ appears to be a common, and possibly critical component of infection-induced miR-155 expression.

Given the critical role of neuroinvasion, and that *L. monocytogenes* infection of macrophages upregulates miR-155 (63), we tested the impact of cellular infection in microglia on upregulation of miR-155 and relevant mRNA's. Infection of SIM-A9 microglial cells with either strain of *L. monocytogenes* upregulated miR-155 (Figure 9C). Strain 10403s appeared to be the more potent stimulator of miR-155, perhaps due to greater induction of *Tnf* (Figure 9D). Genes strongly associated with Type I IFN-response in *L. monocytogenes* infected macrophages and mice, *Ifit3* and *Mx1*, were also upregulated in infected SIM-A9 cells (Figure 9E) (59, 62). These results show *L. monocytogenes* infection of microglia upregulates miR-155 and markers of Type I IFN activation that are also upregulated at 3 d p.i *in vivo*. However, this pattern is not seen *in vivo*, as miR-155 was not upregulated at d 3 p.i. when the probability of bacterial infection is greatest (Figures 4B, 5). Moreover, *in vitro* studies suggest the bacterial load of strain EGD required to upregulate miR-155 in the brain would be much greater than found in this model. Thus, cytokine stimulation seems to be the more likely stimulus *in vivo*.

**Figure 10:**
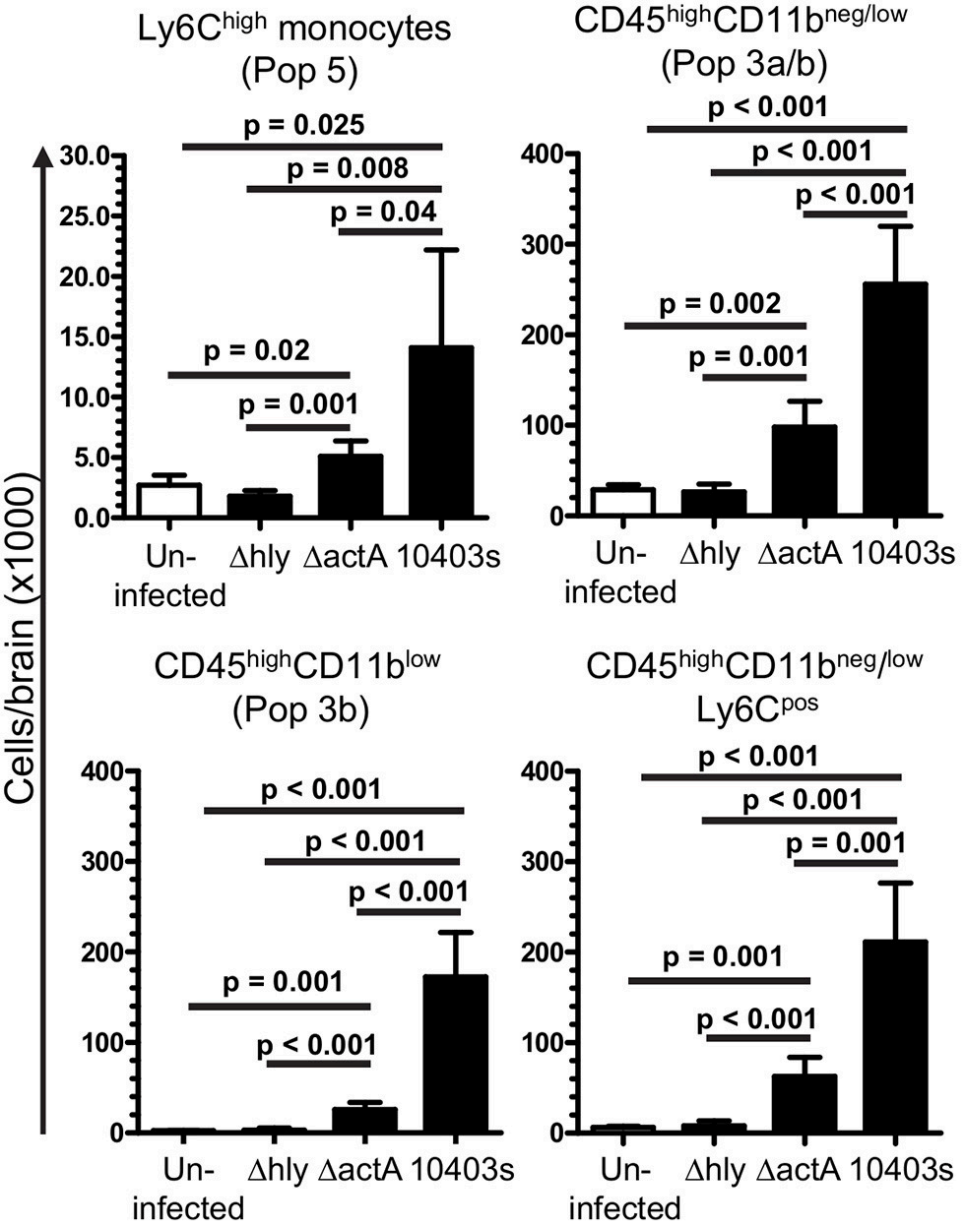
Induction of leukocyte influxes into the CNS by infection with *L. monocytogenes* mutants. B6.WT mice were infected with *L. monocytogenes* strain 10403s (7.0 × 10^5^ CFU), or 10403s-derived Δ*hly* and Δ*actA* mutants (2.0 × 10^7^ CFU), then were treated with antibiotics. Brains were harvested at 7 d p.i. and analyzed by flow cytometry as shown in Figures 4, 5. Results presented are the mean + SD cells/brain from 4 to 6 mice per group. Significant *p*-values calculated by 2-tailed Student's *t*-test are given.

Given the critical role of bone marrow-derived leukocytes as sources of pro-inflammatory cytokines that could upregulate miR-155 (Figure 7), we tested the degree to which *L. monocytogenes* Δ*hly* and Δ*actA* mutants induced influxes of activated cells to the brain. Infection with 10403s and Δ*actA* mutants lead to significant influxes of Ly6C^high^ monocytes and CD45^high^CD11b^neg/low^ cells, including activated CD45^high^CD11b^low^ and Ly6C^pos^CD45^high^CD11b^neg/low^ cells (Figure 10). However, in each case, numbers of recruited cells were significantly greater in 10403s-infected mice than in mice infected with Δ*actA* mutants. In contrast, infection with Δ*hly* mutants caused no measurable leukocyte influxes (Figure 10). These data show that although Δ*actA* mutants induce lymphocyte and monocyte influxes into the brain, and do upregulate *Tnf* (Figure 9B), whatever stimulation they provide to microglia is insufficient in quality or quantity to upregulate miR-155.

**Figure 11:**
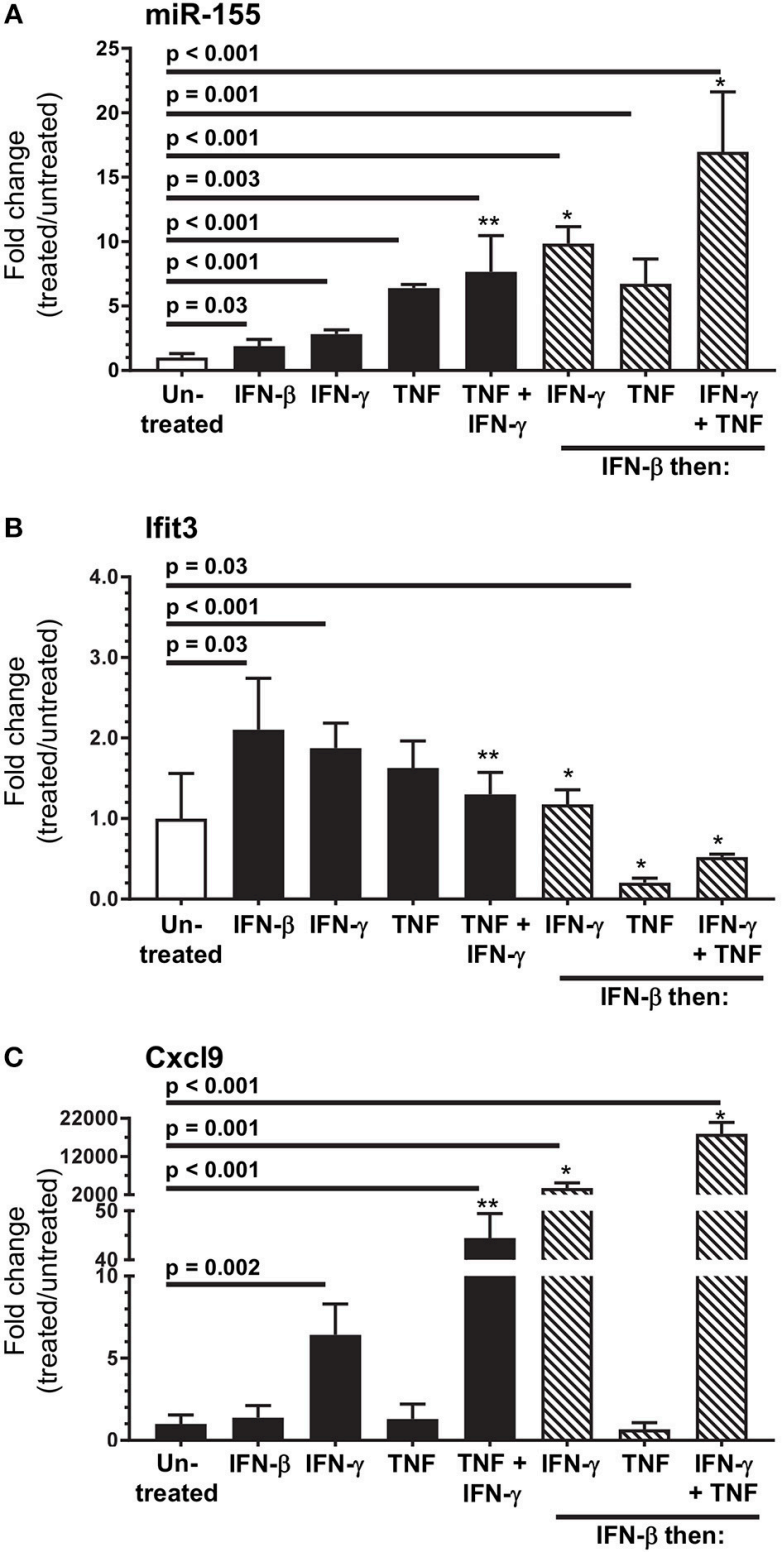
*In vitro* modeling of microglial activation. SIM-A9 cells were stimulated with IFN-β (250 pg/ml), IFN-γ (1.0 ng/ml), TNF (20 ng/ml), or the same concentrations of IFN-γ + TNF for 24 h (black bars), or were incubated with IFN-β (250 pg/ml) for 24 h then washed and incubated another 24 h with the same concentrations of IFN-γ, TNF, and IFN-γ + TNF (hatched bars). Gene expression was measured by qPCR normalized to snoRNA135 for miR-155 **(A)**, or *Gapdh* for *Ifit3* **(B)** and *Cxcl9* **(C)**. Fold changes were calculated against untreated cells (open bars). Significant *p*-values between treated and untreated cells calculated by 2-tailed Student's *t*-test or Mann–Whitney *U*-test are given. ^*^p < 0.05 compared with stimulation with IFN-γ, TNF, or IFN-γ + TNF alone. ^**^p < 0.05 compared with stimulation with IFN-γ alone.

Next we used SIM-A9 cells to model cytokines likely to upregulate miR-155 and activate microglia *in vivo*. Each condition tested upregulated miR-155, and sequential stimulation with IFN-β then IFN-γ or IFN-γ + TNF more robustly upregulated miR-155 than did incubation with only IFN-γ or IFN-γ + TNF (Figure 11A). IFN-β and IFN-γ also upregulated *Ifit3* whereas, none of the other conditions, including IFN-γ + TNF did so (Figure 11B). Interestingly, *Ifit3* expression returned to baseline after IFN-β was removed suggesting stimulation by Type I IFNs was ongoing *in vivo*, at least to 7 d p.i. In contrast, upregulation of *Cxcl9* required stimulation with IFN-γ, either alone or with TNF (Figure 11C). IFN-β and TNF by themselves had no effect on *Cxcl9* expression but both potentiated IFN-γ. Additionally, as found with miR-155, *Cxcl9* expressions in response to IFN-γ and IFN-γ + TNF were enhanced in IFN-β-stimulated cells. Despite challenges estimating physiologic cytokine concentrations and mimicking responses of normal cells, these data show cytokine stimulation could upregulate microglial miR-155. Nonetheless, IFN activation without miR-155 upregulation, as observed *in vivo* at 3 d p.i. (Figures 4B, 5), suggest microglia in the intact brain have a more nuanced response than shown here *in vitro*. These data also support the notion that the microglial response to Type I IFN during the innate host response is supplanted by Type II IFN during the adaptive host response. Additionally, they show that IFN-γ and other cytokines, e.g., TNF, produced by bone marrow-derived cells that enter the brain could act synergistically to stimulate microglia.

### miR-155 inhibits production of pro-inflammatory mediators in microglia

**Figure 12:**
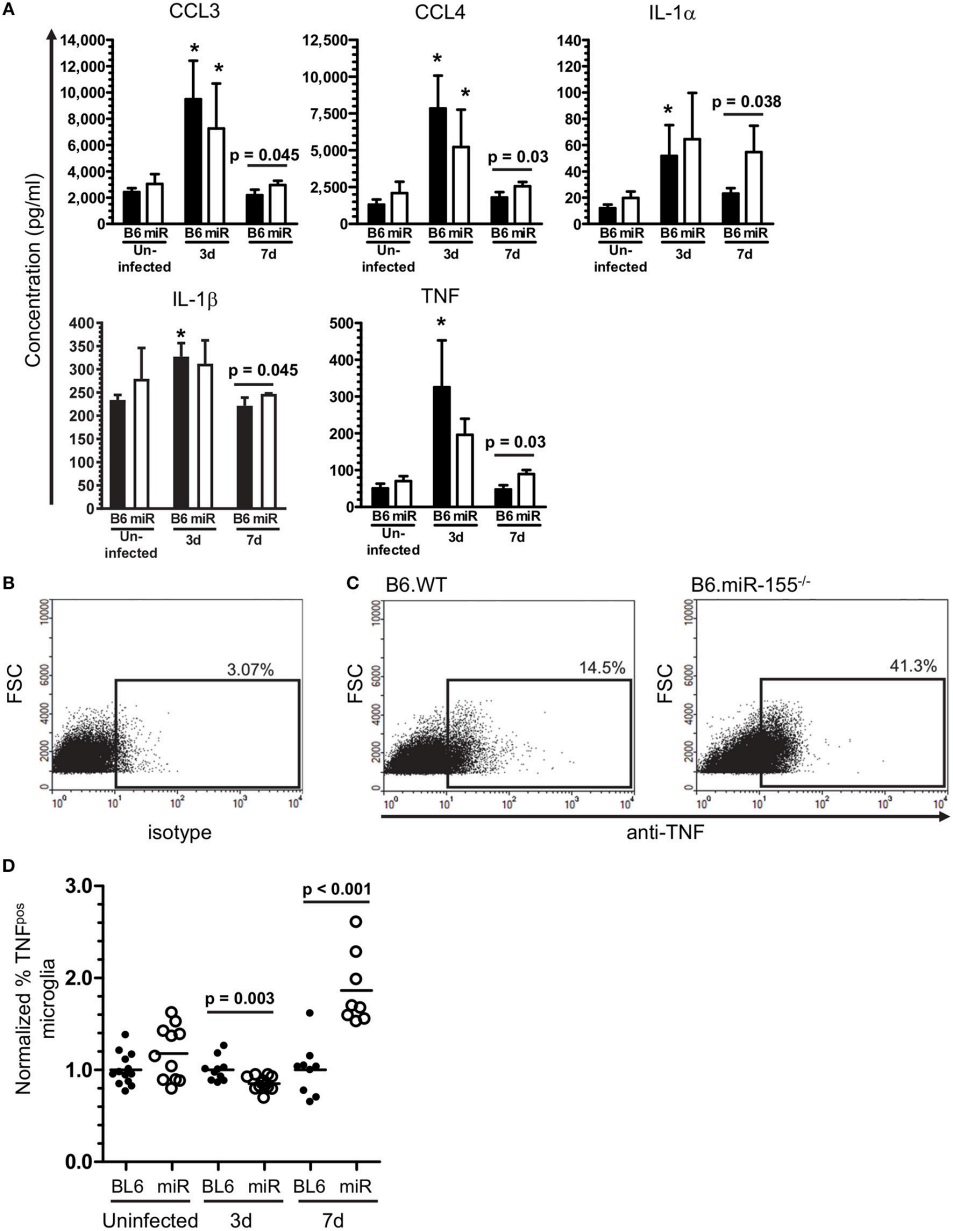
miR-155 inhibits production of pro-inflammatory cytokines after *in vitro* stimulation with heat-killed *L. monocytogenes*. **(A)** CD11b^pos^ brain cells (2.5 × 10^5^/ml) were collected by immunomagnetic sorting from uninfected B6.WT (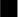) and B6.miR-155^−/−^ (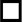) mice and after i.p. infection with 2.1 × 10^5^ CFU *L. monocytogenes* strain EGD, then were incubated overnight with heat-killed *L. monocytogenes* EGD. Mediator concentrations in cell supernatants were measured by bead array and are presented are the mean ± SD concentration (pg/ml) from 4 mice in each genotype/time point. **(B–D)** Brain leukocytes from uninfected mice and from mice infected with 2.1–3.2 × 10^5^ CFU *L. monocytogenes* strain EGD were incubated overnight with heat-killed *L. monocytogenes* EGD, plus Brefeldin A for the last 4–6 h. After immunolabeling of extracellular markers, the cells were fixed and permeabilized then incubated with isotype control or anti-TNF mAb and analyzed by flow cytometry. Microglia were identified as CD45^int^CD11b^pos^ cells. TNF^pos^ microglia were identified in each experiment based on gating with isotype control mAb. Dotplots show TNF expression from representative samples including isotype **(B)**, and 7 d p.i. B6.WT and B6.miR-155^−/−^ **(C)**. Percentages of TNF^pos^ microglia from uninfected mice or mice 3 d and 7 d p.i. were normalized to the mean percentage of cytokine^pos^ microglia in B6.WT mice for that experiment and time post-infection. **(D)** Results from 4 separate experiments are combined and presented as the normalized % TNF^pos^ microglia from uninfected and infected B6.WT (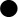) and B6.miR-155^−/−^ (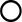) mice, *n* = 8–13 mice from each genotype/time point. Symbols represent individual mice with the horizontal bar at the group mean. Significant *p*-values between genotypes were calculated by 2-tailed Student's *t*-test are given.

To test the role of miR-155 in microglia, CD11b^pos^ brain cells from uninfected and infected B6.WT and B6.miR-155^−/−^ mice were incubated overnight with heat-killed *L. monocytogenes* EGD and concentrations of chemokines and cytokines in supernatants were measured by bead array (Figure 12A). CD11b^pos^ brain cells from B6.WT mice collected at 3 d p.i. produced significantly higher amounts of CCL3, CCL4, IL-1α, IL-1β, and TNF compared with cells from uninfected mice, but without significant differences between genotypes. By 7 d p.i. however, cells from B6.miR-155^−/−^ mice produced significantly higher amounts of these mediators than did cells from B6.WT mice, although with small absolute difference between the genotypes in most cases. The finding of greater concentrations of pro-inflammatory mediators in brain cells from B6.miR-155^−/−^ mice than from B6.WT mice was confirmed in a second experiment using unseparated brain leukocytes instead of CD11b^pos^ brain cells (data not shown). Additionally, TNF production in microglia after overnight incubation with heat-killed *L. monocytogenes* EGD was tested by measuring TNF^pos^ cells by flow cytometry (Figures 12B,C). In each experiment, percentages of TNF^pos^ microglia from each mouse of a particular infection status, i.e., uninfected, 3 and 7 d p.i., were normalized to the mean percentage of cytokine^pos^ microglia in B6.WT mice for that experiment and same infection status (Figure 12D). These results showed nearly 2-fold more microglia from B6.miR-155^−/−^ mice were TNF^pos^ at 7 d p.i. compared with B6.WT mice at 7 d p.i., as well as a slight reduction in TNF^pos^ microglia at 3 d p.i. Similarly, other experiments showed greater intracellular production of IL-β at 7 d p.i. in microglia from B6.miR-155^−/−^ mice (Supplemental Figures 4A–C). Collectively, these data show upregulation of miR-155 in microglia inhibits production of key pro-inflammatory mediators in response to stimulation by heat-killed *L. monocytogenes*.

**Figure 13:**
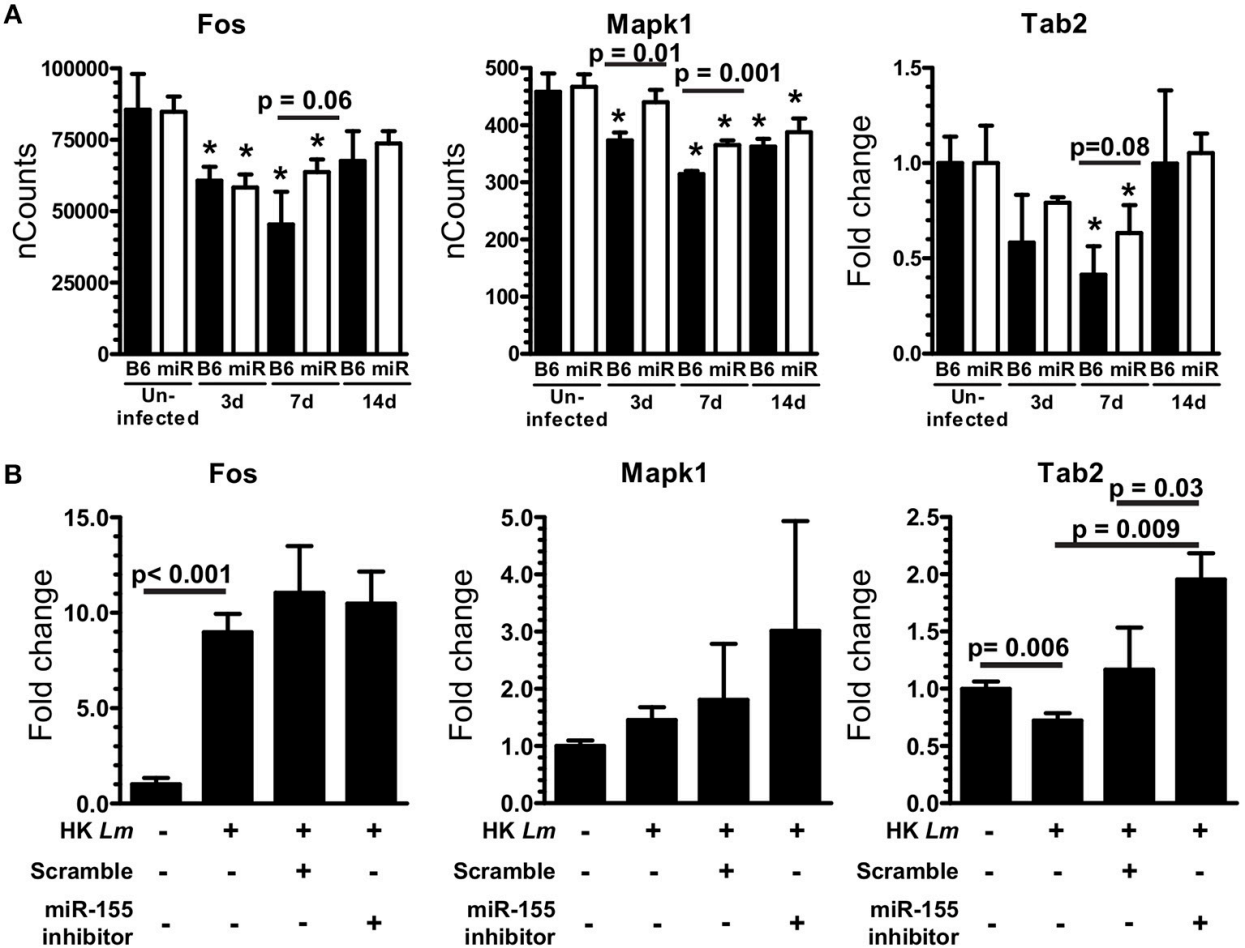
miR-155 targets *Tab2* in microglia**. (A)** Microglia were collected from uninfected and infected B6.WT (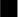) and B6.miR-155^−/−^ (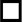) mice described in Figure 6. Expressions of *Fos* and *Mapk1* were measured by nCounts, expression of *Tab2* was measured by qPCR normalized to *Gapdh*. Results presented are the mean ± SD nCounts (*Fos, Mapk1*) or fold change (*Tab2*) compared with uninfected B6.WT from 3 pools of sorted microglia per genotype. **(B)** SIM-A9 microglial cells were transfected with 50 nM miR-155 inhibitor or scramble (control) then were incubated with heat-killed (HK) *L. monocytogenes* for 24 h prior to measuring mRNA expression by qPCR. ^*^p < 0.05 compared with uninfected mice of the same genotype by 1-way ANOVA with Dunnett's post-test. Significant *p*-values between genotypes and cell treatments were calculated by 2-tailed Student's *t*-test.

To identify a mechanism by which miR-155 inhibits pro-inflammatory cytokine production, we evaluated miR-155 target genes included on the NanoString array (Supplemental Table 2) that participate in signaling pathways that upregulate pro-inflammatory mediators. In addition, expression of two relevant miR-155 targets not on the array, *Socs1* and *Tab2*, were measured in the same samples by qPCR. Among these, expressions of *Tab2, Fos*, and *Mapk1*, components of signaling pathways that upregulate TNF, IL-1β, IL-6, CCL3, and CCL4 via AP-1, were increased in microglia from B6.miR-155^−/−^ (Figure 13A). Higher expression of these genes in B6.miR-155^−/−^ microglia suggests they could be targeted by miR-155 to inhibit inflammatory responses (32). In contrast, *Socs1* was not changed in microglia from B6.miR-155^−/−^ mice when compared with B6. WT mice (Supplemental Figure 3). Subsequently, miR-155 was knocked-down in SIM-A9 cells to test for increased expression of putative target mRNAs (*Fos, Mapk1*, and *Tab2*) after stimulation with heat-killed *L. monocytogenes* EGD (Figure 13B). miR-155 inhibitor significantly increased expression of *Tab2*, but not that of *Fos* or *Mapk1*. Thus, targeting of *Tab2* is another potential mechanism by which miR-155 can alter microglial responses during brain infection.

## Discussion

**Figure 14:**
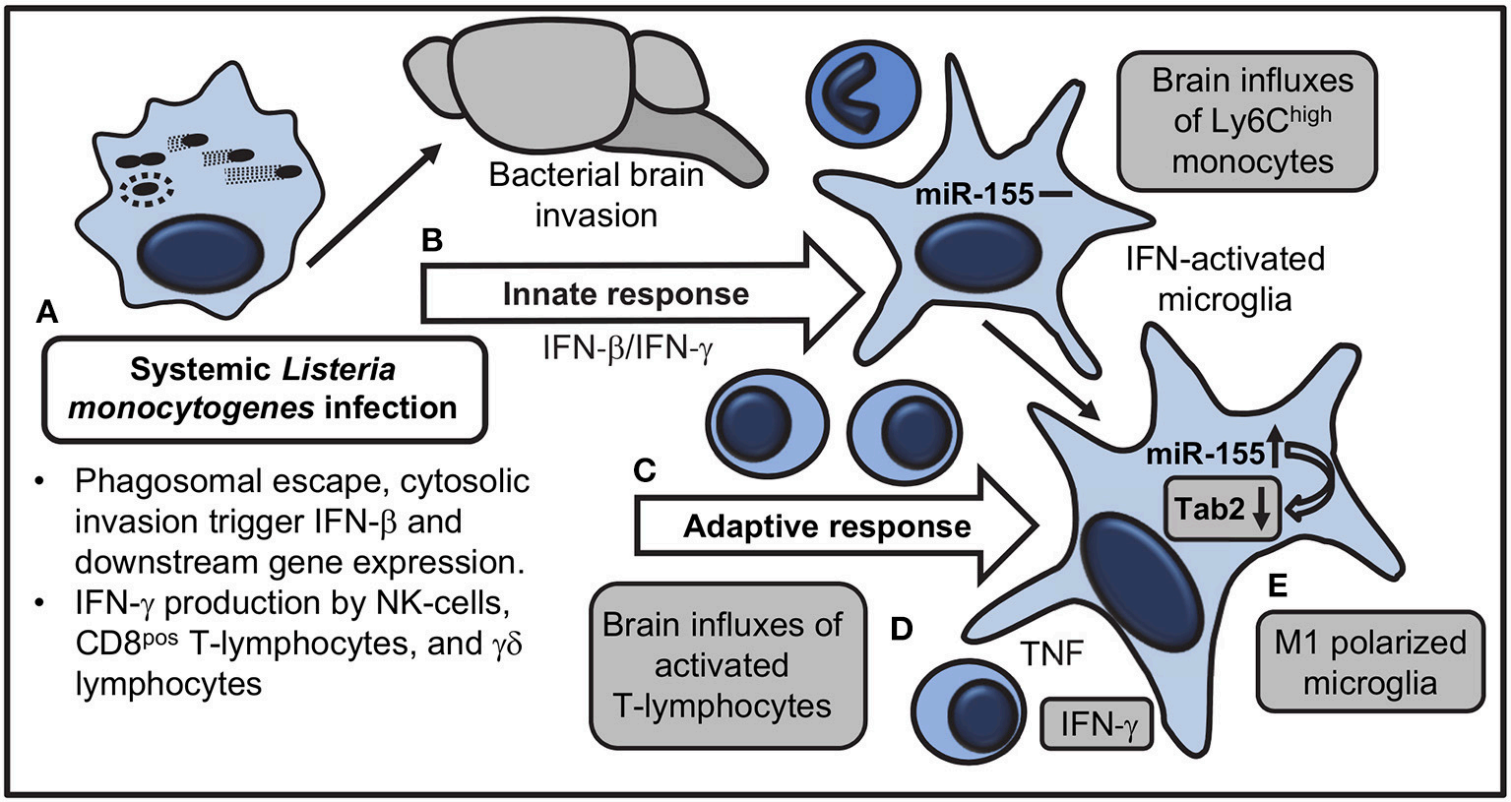
Model for actions of miR-155 that influence brain inflammation and microglial activation during systemic *L. monocytogenes* infection. **(A)** Systemically injected bacteria escape from phagosomes and trigger transcription of *Ifnb* and downstream genes, as well as IFN-γ production by NK-cells, CD8 T-lymphocytes, and γδ T-cells. **(B)** Innate responses trigger brain influxes of Ly6C^high^ monocytes and IFN-activation of microglia without upregulation of miR-155. Bacteria invade the brain. **(C)** IFN-γ-secreting T lymphocytes expand during the adaptive immune response and are recruited to the brain. **(D)** Pro-inflammatory cytokines, in particular IFN-γ, produced by recruited leukocytes stimulate microglia. **(E)** miR-155 and IFN-γ-related genes are upregulated in microglia during M1 polarization. miR-155 targets mRNA for the adapter molecule *Tab2* to downregulate TLR-mediated cytokine production. Shaded boxes show processes altered in B6.miR-155^−/−^ mice.

Manipulating miR expression is a promising means for controlling neuro-inflammation (13, 66). Experiments here analyzed expression and function of miR-155 in brains of mice in a treatment model of lethal neuroinvasive *L. monocytogenes* infection. This is important because case fatality rates of bacterial meningitis have improved only modestly over the past four decades (67, 68), and neurological complications are common in up to a third of survivors (69). Thus it is important to study survivors of CNS infection to identify means for improving neurological function. These studies show miR-155 is upregulated in the brains of infected mice, and exerts pro- and anti-inflammatory actions by at least three different mechanisms (Figure 14). Systemic miR-155 enhances brain inflammation through its required role in optimal development of IFN-γ-secreting lymphocytes (34, 35), cells that enter the brain and activate microglia during the adaptive immune response. In addition, microglial miR-155 promotes M1 polarization and also inhibits inflammatory responses to TLR stimulation, the latter likely by targeting *Tab2*.

*L. monocytogenes* Δ*actA* and Δ*hly* mutants were used to test the extent to which bacterial neuroinvasion was required to upregulate miR-155. Internalized Δ*actA* mutants lyse phagosomes then enter and replicate within the cytosolic compartment, but cannot spread cell-to-cell due to a lack of motility [reviewed in (64)]. In contrast, Δ*hly* mutants are retained within phagosomes and killed. Importantly, Δ*actA* but not Δ*hly* mutants activate cytosolic surveillance mechanisms that induce IFN-β, nuclear translocation of NF-κB, and upregulation of their downstream genes (62, 70). Although neither mutant invades the CNS, systemic responses initiated by Δ*actA* mutants trigger immune response genes in the brain and stimulate lymphocyte and Ly6C^high^ monocyte influxes to the brain (44, 46). Results presented here showed that neither mutant upregulated brain miR-155 even though both upregulated TNF mRNA in the brain, and Δ*actA* mutants also induced a degree of leukocyte influxes.

Despite this essential role for neuroinvasion, and the fact that *L. monocytogenes* can upregulate miR-155 in SIM-A9 microglial cells (this work) and in macrophages (63), other experiments suggested microglial infection was not a likely stimulus *in vivo*. For example, miR-155 was not upregulated at d 3 p.i. when infection is most likely in this model. In addition, previous data showed 95% of *L. monocytogenes*-infected CD11b^pos^ cells in brains of systemically infected mice were Ly6C^high^ monocytes or neutrophils, indicating few microglia were infected (71). Furthermore, *in vitro* studies presented here suggested a higher bacterial load, i.e., CFU/brain, was required to induce miR-155 expression than is achieved in the model. Thus, cytokine stimulation is the most plausible trigger of miR-155 expression. Nonetheless, neuroinvasion could lead to blood-brain barrier breakdown or engender interactions between infiltrating bone marrow-derived cells and microglia that are necessary for inducing miR-155 (61). This could explain why infection with *L. monocytogenes* Δ*actA* and Δ*hly* mutants could induce *Tnf* expression, and in the case of Δ*actA* mutants also trigger influxes of activated lymphocytes, without upregulating miR-155. Another finding was that neuroinvasive but not non-neuroinvasive *L. monocytogenes* upregulated *Ifng* expression, suggesting it also has a required *in vivo* role.

Systemic *L. monocytogenes* infection induces types I, II, and III IFNs resulting in a prominent IFN signature in blood, spleen, and liver during the first 3 days of infection (59, 72). In accord, our results at 3 d p.i. show upregulation of IFN-related genes in microglia during the innate response to systemic infection. The gene expression profile at this time suggested microglia are primarily sensing Type I rather than Type II IFNs even though both are produced in the periphery at this time (59, 72), and gene expression in whole brain specimens and recruitment of Ly6C^high^ monocytes are notably impaired in IFN-γ^−/−^ mice (46, 73). Upregulation of miR-155 in microglia by Type I IFN *in vitro* found here and in bone marrow-derived macrophages contrasts with *in vivo* findings at 3 d p.i. (17). This could be a function of different cells, a dose response, or is due to the regulatory environment of the intact brain (74). By 7 d p.i., microglia showed clear evidence of IFN-γ stimulation, including upregulation of miR-155 and M1 polarization, likely due to cytokines produced by activated lymphocytes recruited to the brain. This finding agrees with studies showing *L. monocytogenes* infection rapidly expands IFN-γ-secreting CD8^pos^ T-lymphocytes which, along with other IFN-γ-secreting cells, are recruited into the brain with peak frequencies around 7 d p.i. (44, 75, 76).

Comparison of gene expression in microglia of infected B6.WT and B6.miR-155^−/−^ mice suggested a key action of miR-155 in the brain is actually through its required role for optimal development of IFN-γ secreting CD8^pos^ T-cells (34, 35). Our studies were limited by not measuring IFN-γ production in brain lymphocytes in response to antigen-specific stimulus. Nonetheless, B6.miR-155^−/−^ mice had lower expression of IFN-related genes in microglia at 7 d p.i., fewer IFN-γ transcripts in bone marrow-derived cells isolated from their brains, and reduced influxes of activated CD3^pos^ T-lymphocytes. Recruitment of Ly6C^high^ monocytes was somewhat reduced in B6.miR-155^−/−^ mice at 7 d p.i., but differences between B6.miR-155^−/−^ and B6.WT mice were not as robust as for lymphocyte recruitment. Although not tested for specifically, reduced IFN-γ stimulation of microglia in B6.miR-155^−/−^ mice and subsequently diminished expression of chemokines such as CXCL9 and CXCL10, as well as other components of leukocyte migration not tested for, e.g., endothelial adhesion molecules, resulted in reduced leukocyte recruitment. Such a scenario has been shown in other models of CNS infection (77–79).

A second pro-inflammatory action of miR-155 is through promotion of M1 polarization (11). Microglia in B6.miR-155^−/−^ mice were activated but not fully polarized compared with microglia from B6.WT mice. In addition, *in vitro* incubation of cells with heat-killed *L. monocytogenes* revealed miR-155 may also exert an anti-inflammatory role in microglia. In this situation cells from B6.miR-155^−/−^ mice produced more pro-inflammatory cytokines and chemokines at 7 d p.i. than did cells from B6.WT mice. This result could be due to miR-155 targeting *Tab2*, as shown in human dendritic cells (32) and human CHME3 microglial cells infected with Japanese encephalitis virus (80). Although our *in vivo* data suggested miR-155 contributed to *Tab2* downregulation in microglia during infection, they also showed it was not solely responsible for this finding. Collectively, analysis of microglia and brain inflammation in B6.miR-155^−/−^ mice indicated that loss of miR-155's inhibitory action was overshadowed by the more robust impact of reduced IFN-γ stimulation and muted microglial polarization.

Collectively, these results show that there are multiple mechanisms by which miR-155 modulates CNS inflammation during neuroinvasive *L. monocytogenes* infection (Figure 14). Given the many targets of miR-155, other mechanisms by which it could ameliorate CNS pathology during neuroinvasive infection are also possible. Data presented here support further study of manipulating expression of miR-155 and its downstream targets as adjunctive anti-inflammatory therapy during neuroinvasive bacterial infection.

## Author contributions

DD, MZ, and AG developed the project, secured funding designed, and performed research, analyzed data and wrote the paper. BC, JG, JN, JS, and MB designed and performed research and analyzed data. JS provided expert statistical analysis. All authors read and approved the final manuscript.

### Conflict of interest statement

The authors declare that the research was conducted in the absence of any commercial or financial relationships that could be construed as a potential conflict of interest.
