# Supplementary material for: Neuroinvasive Listeria monocytogenes Infection Triggers IFN-Activation of Microglia and Upregulates Microglial miR-155
Source: Front Immunol. 2018 Nov 27;9:2751. doi: 10.3389/fimmu.2018.02751 (PMC6277692; doi:10.3389/fimmu.2018.02751)
Supplement: Supplementary file 1 [file Table_1.DOCX]

**Table 1. Primers used for quantitative real-time PCR.**

| **Gene** |  | **Primer Sequence (5' - 3')** | **PrimerBank ID/ Source** |
| --- | --- | --- | --- |
| Cxcl9 | Forward  Reverse | GGAGTTCGAGGAACCCTAGTG  GGGATTTGTAGTGGATCGTGC | 162287427c1 |
| Fos | Forward  Reverse | CGGGTTTCAACGCCGACTA  TTGGCACTAGAGACGGACAGA | 6753894a1 |
| Ifit3 | Forward  Reverse | CCTACATAAAGCACCTAGATGGC  ATGTGATAGTAGATCCAGGCGT | 6754288a2 |
| Ifng | Forward  Reverse | GGAGGAACTGGCAAAAGGATG  AGGTGTGATTCAATGACGCTTATG | 33468859a1 |
| Mapk1 | Forward  Reverse | GGTTGTTCCCAAATGCTGACT  CAACTTCAATCCTCTTGTGAGGG | 33090821a1 |
| Mx1 | Forward  Reverse | GACCATAGGGGTCTTGACCAA  AGACTTGCTCTTTCTGAAAAGCC | 6996930a1 |
| Socs1 | Forward  Reverse | CTGCGGCTTCTATTGGGGAC  AAAAGGCAGTCGAAGGTCTCG | 6753424a1 |
| Tab2 | Forward  Reverse | CATGACCTGCGACAAAAATTCC  TGATTGCGTAGACCAGAAATTCC | 20149752a1 |
| Tnf | Forward  Reverse | CCACGCTCTTCTGTCTACTG  ATCTGAGTGTGAGGGTCTGG | 133892368c1 |
| Il1b | Forward  Reverse | TGACGGACCCCAAAAGATGAAG  GTGATACTGCCTGCCTGAAGC | Beacon Designer |
| Gapdh | Forward  Reverse | GGCAAATTCAACGGCACAGT  AGATGGTGATGGGCTTCCC | Wilkinson et al., 2011 |
