# Supplementary material for: Neuroinvasive Listeria monocytogenes Infection Triggers IFN-Activation of Microglia and Upregulates Microglial miR-155
Source: Front Immunol. 2018 Nov 27;9:2751. doi: 10.3389/fimmu.2018.02751 (PMC6277692; doi:10.3389/fimmu.2018.02751)
Supplement: Supplementary file 2 [file Table_2.DOCX]

**Table 2. miR-155 targets covered on the NanoString Inflammation v2 mouse array.** Gene targets listed on the NanoString Inflammation v2 mouse array were queried in the indicated public databases to assess their association with miR-155.

| **Gene** | **Cm.jefferson.org** | **MIRDB** | **miRTarbase** | **TargetScan7.1** | **Gaudet et al** |
| --- | --- | --- | --- | --- | --- |
| Bcl6 |  |  |  |  | x |
| Ccl20 | x |  |  |  |  |
| Ccr7 |  |  |  |  | x |
| Cd163 | x |  |  |  |  |
| Cebpb |  | x | x | x |  |
| Creb1 | x |  |  | x |  |
| Csf3 | x |  |  |  |  |
| Fos |  |  | x |  |  |
| Gnas |  |  |  | x |  |
| Hdac4 |  |  | x |  |  |
| Hif1a | x | x |  |  |  |
| Hmgb1 | x |  |  |  |  |
| Ifit1 | x |  |  |  |  |
| Il1rap | x |  |  |  |  |
| Il21 | x |  |  | x |  |
| Il6ra |  | x | x |  |  |
| Mapk1 |  |  | x |  |  |
| Mrc1 |  |  |  |  | x |
| Mef2a | x |  | x | x |  |
| Nfe2l2 |  | x |  | x |  |
| Nlrp3 | x |  |  |  |  |
| Nos2 | x |  |  |  |  |
| Rela | x | x |  | x |  |
| Rhoa |  |  | x |  |  |
| Ripk1 |  |  | x |  |  |
| Rock2 | x |  |  |  |  |
| Rps6ka5 |  | x |  |  |  |
| Ptger2 | x |  |  |  |  |
| Ptger4 | x |  |  |  |  |
| Ptgfr | x |  |  |  |  |
| Tgfbr1 | x |  |  |  |  |
