# Supplementary material for: Neuroinvasive Listeria monocytogenes Infection Triggers IFN-Activation of Microglia and Upregulates Microglial miR-155
Source: Front Immunol. 2018 Nov 27;9:2751. doi: 10.3389/fimmu.2018.02751 (PMC6277692; doi:10.3389/fimmu.2018.02751)
Supplement: Supplementary file 4 [file Data_Sheet_1.PDF]

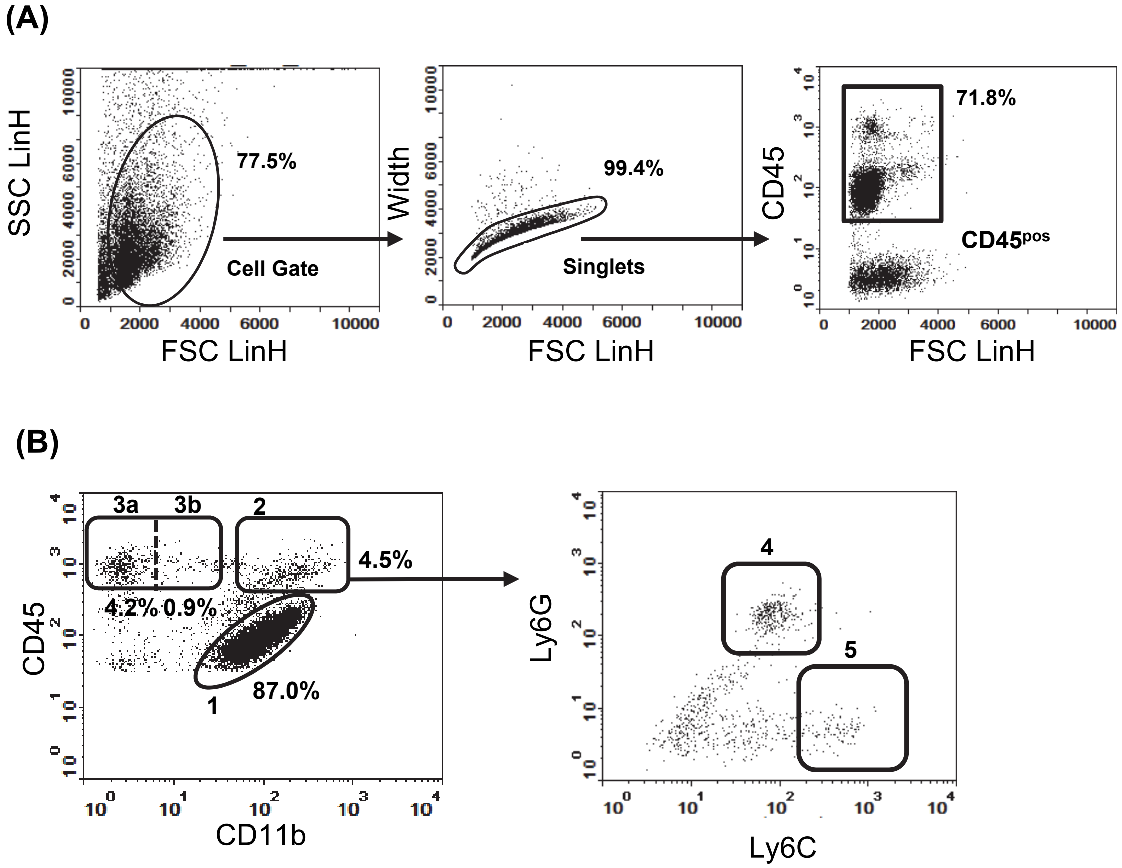

Supplemental Figure 1. Gating strategy for analysis of brain leukocytes. Cells from enzymatically digested brains were centrifuged through 30% Percoll to remove myelin. Erythrocytes were lysed then cells were incubated with mAb and analyzed by flow cytometry. (A) Dotplot shows total brain cells gated to exclude debris (Cell Gate) and cell doublets (Singlets) followed by selection of CD45^{pos} cells. (B) Gated CD45^{pos} cells are divided into CD45^{int}CD11b^{pos} microglia (1), CD45^{high}CD11b^{high} leukocytes (2) and CD45^{high}CD11b$^{neg/low}$ leukocytes (3). Gated CD45^{high}CD11b^{high} leukocytes reveal Ly6G^{pos} neutrophils (4) and Ly-6C^{high} monocytes (5), whereas CD45^{high}CD11b$^{neg/low}$ leukocytes are sub-divided into CD11b^{neg} (Pop. 3a) and CD11b^{low} cells (Pop. 3b).

(A)

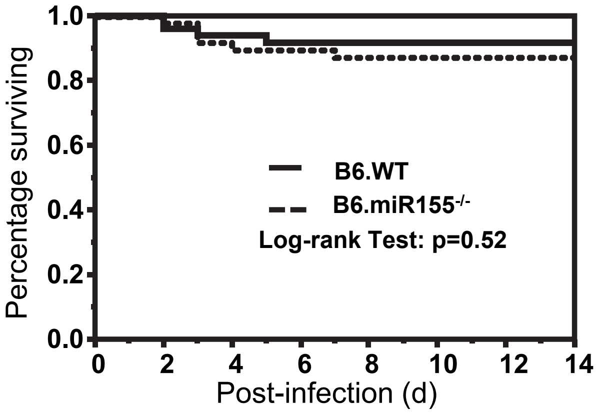

(B)

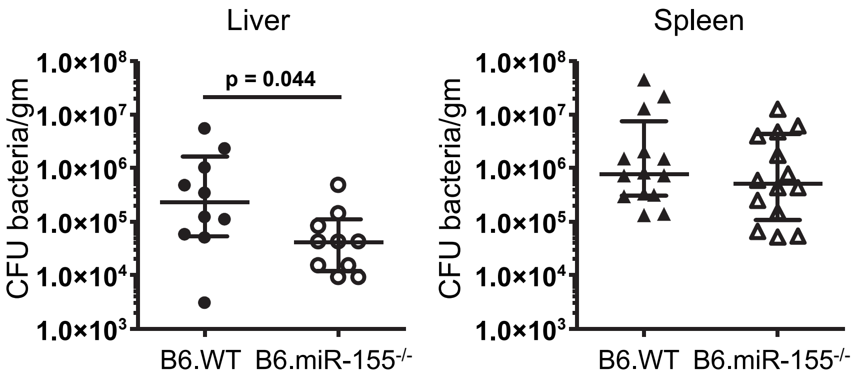

Supplemental Figure 2. Survival of antibiotic-treated B6.WT and B6.miR-155$^{-/-}$ mice following lethal *L. monocytogenes* infection. Age and sex-matched B6.WT and B6.miR-155$^{-/-}$ mice were infected with *L. monocytogenes* EGD and treated with antibiotics. Animals were considered to have succumbed to infection if they were found dead or required euthanasia prior to intended date of harvest. (A) Time to death distributions were estimated using the Kaplan-Meier method and results between the B6.WT (n=50, 25M, 25F) and B6.miR-155$^{-/-}$ (n=50, 25M, 25F) mice were compared using a log-rank test. Results are from combined from 6 separate injections in which mice received a (mean ± SD) inoculum of 3.4 ± 0.33 x 10^5 CFU bacteria. (B) Bacterial loads in liver and spleen 3d p.i. were measured by serial dilution and plating on agar. Results from individual female B6.WT (●,▲) and B6.miR-155$^{-/-}$ (○,△) mice are presented as the median ± IQR CFU bacteria/gm tissue of 10 mice/genotype (liver) and 14 mice/genotype (spleen). Statistical analysis was performed using the Mann-Whitney test.

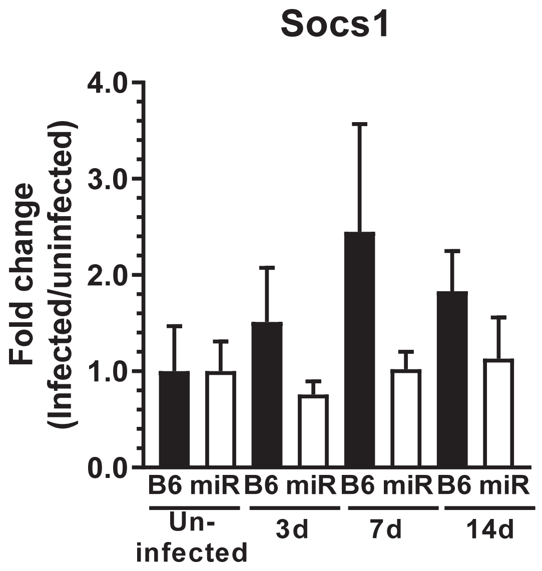

Supplemental Figure 3. Expression of *Socs1* in microglia. Microglia were collected from uninfected and infected B6.WT (■) and B6.miR-155$^{-/-}$ (□) mice described in Fig. 5. Expressions of *Socs1* was measured by qPCR normalized to *Gapdh*. Results presented are the mean ± SD of fold change compared with uninfected B6.WT from 3 pools of sorted microglia per genotype.

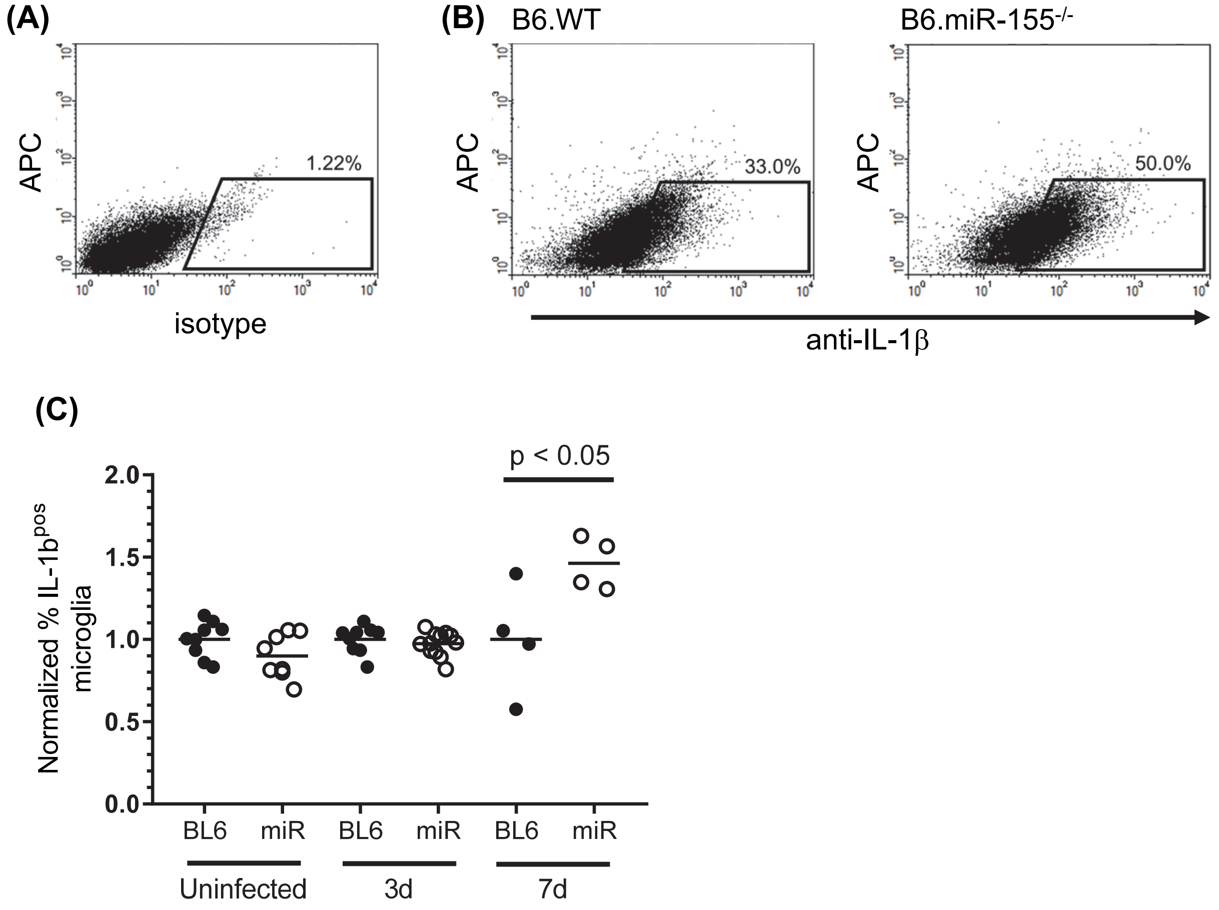

Supplemental Figure 4. Microglia from B6.miR-155$^{-/-}$ mice have increased production of IL-1β after stimulation with heat-killed *L. monocytogenes*. Brain leukocytes from uninfected mice and from mice infected with 2.1 – 3.3 x 10^5 CFU *L. monocytogenes* strain EGD were incubated overnight with heat-killed *L. monocytogenes* EGD, plus Brefeldin A for the last 4-6hrs. After immunolabeling of extracellular markers, the cells were fixed and permeabilized then incubated with anti-IL-1β or isotype control mAb and analyzed by flow cytometry. Microglia were identified as $CD45^{int}CD11b^{pos}$ cells. (A&B) IL-β^{pos} microglia were identified in each experiment based on gating of mixed B6.WT and B6.miR-155$^{-/-}$ cells labeled with isotype control mAb (A). Dotplots in (B) show representative B6.WT and B6.miR-155$^{-/-}$ cells at 7d p.i. Percentages of IL-β^{pos} microglia from uninfected mice or mice 3d and 7d p.i. were normalized to the mean percentage of cytokine^{pos} microglia in B6.WT mice for that experiment and time post-infection. (C) Results from 5 separate experiments are combined and presented as the normalized % IL-β^{pos} microglia from uninfected and infected B6.WT (●) and B6.miR-155$^{-/-}$ (O) mice, n= 4-12 mice from each genotype/time point. Symbols represent individual mice with the horizontal bar at the group mean. Significant p values between genotypes were calculated by 2-tailed Student's t-test are given.
